# Unique microbial communities and phylosymbiosis signals in herpetofauna

**DOI:** 10.1093/ismejo/wrag076

**Published:** 2026-04-02

**Authors:** Jiaying Li, Yuze Gao, Xuelin Zhao, Jiahao Zhu, Si Zheng, Qihan Guo, Longhui Zhao, Guocheng Shu, Yuzhou Gong, Wujie Xu, Ting Chen

**Affiliations:** Department of Automation, Tsinghua University, Beijing 100084, China; Department of Computer Science and Technology, Tsinghua University, Beijing 100084, China; School of Marine Sciences, Ningbo University, Ningbo 315211, China; Department of Computer Science and Technology, Tsinghua University, Beijing 100084, China; Department of Computer Science and Technology, Tsinghua University, Beijing 100084, China; Institute of Medical Information, Chinese Academy of Medical Sciences and Peking Union Medical College, Beijing 100020, China; Department of Computer Science and Technology, Tsinghua University, Beijing 100084, China; Ministry of Education Key Laboratory for Ecology of Tropical Islands, Key Laboratory of Tropical Animal and Plant Ecology of Hainan Province, College of Life Sciences, Hainan Normal University, Haikou, Hainan 571158, China; Faculty of Agriculture, Forest and Food Engineering, Yibin University, Yibin 644000, China; College of Fisheries, Hunan Agricultural University, Changsha 410128, China; Yuelushan Laboratory, Changsha 410128, China; South China Sea Fisheries Research Institute, Chinese Academy of Fishery Sciences, Guangzhou 510300, China; Department of Computer Science and Technology, Tsinghua University, Beijing 100084, China; State Key Laboratory of Complex, Severe, and Rare Diseases, Peking Union Medical College Hospital, Beijing 100730, China; Department of Bioinformatics, Fujian Key Laboratory of Medical Bioinformatics, Institute of Precision Medicine, School of Medical Technology and Engineering, Fujian Medical University, Fuzhou 350122, China

**Keywords:** symbiotic microbiota, host phylogeny, phylosymbiosis, herpetofauna

## Abstract

Microbial symbionts are closely related to the internal and external factors of their host. However, the prevalence of phylosymbiosis (the presence of host phylogenetic signal in microbial community composition) remains controversial, especially in animals collectively referred to as herpetofauna. To expand our understanding of host-microbiota interactions, we analysed 11 697 symbiotic microbiota samples from of 337 herpetofaunal species, covering skin, oral cavity, gut, cloaca, feces, and other body sites. The composition of the microbial communities gradually changes along the digestive tract, and is host-specific in each region. Overall, herpetofauna’s dominant microbial taxa (*Firmicutes*, *Proteobacteria*, *Bacteroidota*) are more similar to mammals than fish (which are dominated by *Proteobacteria*, *Firmicutes*, and *Fusobacteriota*). However, phylosymbiosis in herpetofauna is weaker than in mammals and tends to occur at higher host taxonomic levels. The strength of the phylosymbiosis signal is influenced by body site, host genetic distance, and analytical method. It indicates that phylosymbiosis exists but is not universal. The intensity and significance of this signal are influenced by host taxonomic scale, the location of the microbial communities, and the assessment methods. These results advance our knowledge of host–microbe interactions across the Tree of Life.

Assembly of symbiotic microbial communities is influenced by both internal factors and external factors. Clarification of phylosymbiosis (the microbial community harbors a phylogenetic signal of the host) is necessary to unravel host–microbiota interactions. A large number of studies on phylosymbiosis have been conducted across diverse lineages and body sites of mammals [1–5]. However, research into phylosymbiosis in birds, reptiles, amphibians, and fish is insufficient and conflicting viewpoints exist. Thus, whether phylosymbiosis is universal remains unclear [6–12].

Amphibians and reptiles (i.e. herpetofauna) occupy crucial positions in the evolution of tetrapods, and are often studied together due to similar physiological and ecological characteristics [13, 14]. Previous studies have revealed the roles of internal and external factors in shaping herpetofaunal microbia [15–18]. Osborne et al. (2024) and Zhu et al. (2025) proposed significant phylosymbiotic signals in herpetofauna [12, 19], whereas Bletz et al. (2017) and Hoffbeck et al. (2023) arrived at opposite conclusions [11, 20]. Focusing on few target host species and/or single body sites is insufficient to investigate phylosymbiosis in herpetofauna. Several critical questions remain unanswered: What are the similarities and differences between herpetofaunal microbiota and that of other vertebrates? How does the strength of host phylogeny’s influence vary across body sites? Do microbial communities reflect host phylogeny?

To address unanswered questions, we constructed an online database of herpetofaunal microbiota (HMicrodb, https://herpdb.com/) comprising 11 697 microbial samples from 337 host species across various body sites (including skin, oral cavity, stomach, gut, cloaca, feces, and other body sites) (Fig. 1A and B; see Supplementary materials and methods and Supplementary Tables S1, S2) [21]. Comprehensive analysis of this dataset found that both body site and host species influenced microbial diversity, composition, and potential functions (predicted using Picrust2 software, see Supplementary materials and methods, See Supplementary materials and methods. Figure 1C–F; Fig. 2A; Supplementary Figs S1–S6; Supplementary Tables S3–S9). Consistent with fish and mammals [22, 23], significant variations in microbial diversities were observed among different herpetofaunal body sites (*P* < 0.01). Adjacent region harbored similar microbial compositions, with similarity decreasing as distance increased (Fig. 1F). This indicates that exogenous microorganisms can spread from the oral cavity along the digestive tract to other areas through the nutrient flow. The growth of these microorganisms is restricted by different host-derived factors (such as oxygen level, pH, antimicrobials) in different regions of the gastrointestinal tract [24].

**Figure 1:**
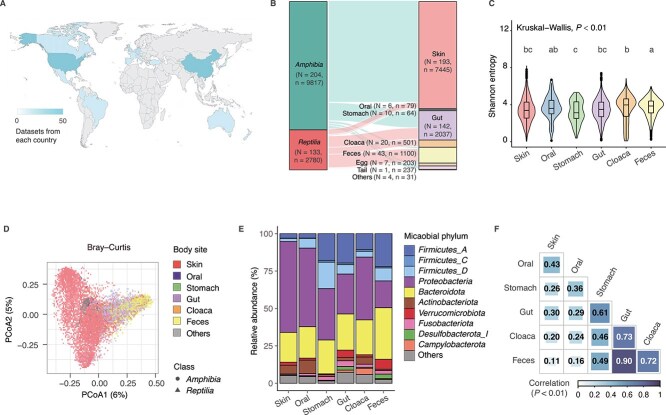
The diversity, composition and phylosymbiosis patterns of microbiota across multiple body sites in herpetofauna. (A) Geographic location of sample collection. Location was obtained from NCBI SRA biosample records or the associated manuscript. Colours represent the number of datasets from each location, with dark blue the most and light blue the least. (B) Sankey plot displaying the distribution of host taxonomy, sample size and related body sites involving in this study. The uppercase “N” indicates the number of host species, and the lowercase “n” indicates the number of samples. The category “Others” includes esophagus, oviduct, pelvis, lumina, and mucosa. (C) Alpha diversity of Shannon entropy at genus level in samples grouped by host body sites. Different letters above box plots indicate significant differences between body sites, respecitively (P < 0.05). (D) Beta diversity measured by principal coordinates analysis (PCoA) based on Bray–Curtis distance for microbial community at genus level. Each point was colored by the corresponding sample belonged host body site and shaped by host class. The category “Others” includes esophagus, oviduct, pelvis, lumina, mucosa, egg, and tail. The outliers were not shown. PERMANOVA results for the effects of body site, host species and their interaction are shown in Supplementary Table S4. (E) Abundances of the major microbial phyla across different body sites. (F) Pearson’s correlation based on the relative abundance of microbiota at genus level among each body sites.

**Figure 2:**
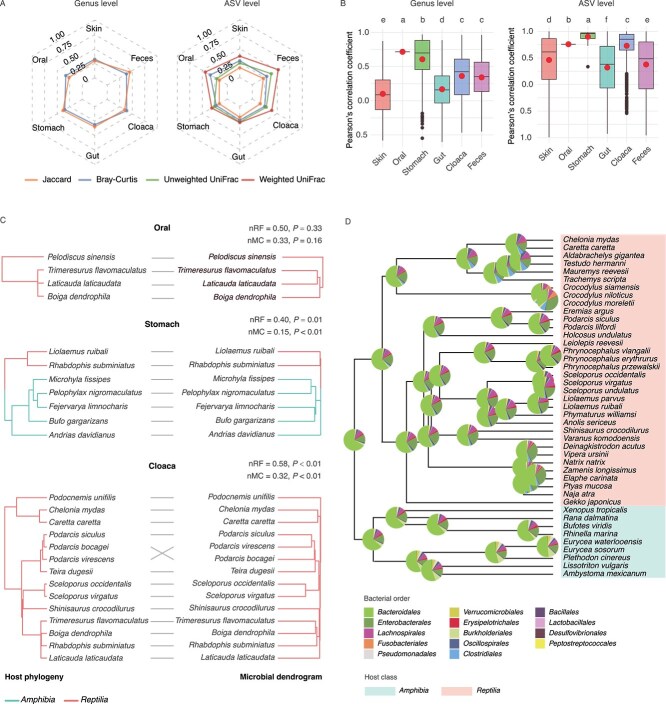
The relationship between host phylogeny and the microbial community across different body sites. (A) The radar graph shows the effects of the host species on the beta diversity of the microbial community at the genus and ASV levels, as estimated by PERMANOVA. The effect size has been standardized by calculating partial omega-squared. The *P* values for all groups were less than 0.01. (B) The boxplot shows the distribution of the correlation coefficients obtained through Mantel test between the host phylogenetic distance matrix and the microbial beta diversity matrix based on Jaccard distance metrics at genus level and ASV level. Host species at each body site were resampled to a uniform size (determined by the oral cavity, the site with fewest species). Correlations were calculated via sub-matrices (999 permutations), with 10 000 random samplings per body site dataset. The red point marks the mean correlation of each body site. Thicker dots represent outlier values. Different letters above box plots indicate significant differences between body sites, respecitively (*P* < 0.05). (C) The host species phylogenetic tree (left) and the symbiotic microbiota dendrogram based on Jaccard distance metrics at ASV level (right). Nomalized Robinson–Foulds (nRF) and Nomalized Matching Cluster (nMC) scale from 0.0 (complete congruence) to 1.0 (complete incongruence). (D) Ancestral reconstruction of feces microbiota with phylogenetic tree of the host species and associated relative abundances of the 14 most abundant bacterial orders. The pie charts at the root and nodes of the tree show the estimated ancestral microbiota compositions (the mean of the posterior distribution at the root, and the generalized least squares estimates at the other internal nodes).

The symbiotic microbiota of herpetofauna was dominated by *Firmicutes*, *Proteobacteria* and *Bacteroidota* (Fig. 1E), which is more similar to the composition of mammals than fish (the main microbial community consists of *Proteobacteria*, *Firmicutes* and *Fusobacteriota*) (See Supplementary materials and methods, Supplementary Fig. S7, and Supplementary Table S1) [22, 25]. For each body sites, the microbial communities or potential functions are host–specific, with host species accounting for 16%–45% of the variation (Fig. 2A, Supplementary Table S10).

To identify phylosymbiosis signals, we analysed the relationships between host phylogeny and the symbiotic microbiota across 267 of these host species using methods based on matrix similarity, topological structure consistency, and multivariate Brownian motion model (see Supplementary materials and methods). Microbial taxa variation attributable to host phylogeny ranged from < 0.01% to 76% (Supplementary Figs S9–S14). Overall, the phylosymbiosis in herpetofauna tends to be more evident on a large scale than on a fine scale (where normalized Matching Cluster (nMC) score is usually smaller than normalized Robinson-Foulds (nRF) score for the same body site), which aligns with previous studies on mammals [4, 26, 27]. Phylosymbiosis signal is the strong and stable in the stomach (Fig. 2B–D, Supplementary Figs S15–S27, Supplementary Tables S11–S22). This might be due to the selective pressure exerted by the acidic environment and digestive enzyme composition of different host lineages, resulting in the selection of conserved acid-resistant microbiota as core taxa (strengthening the matching across large host taxonomic scales). Meanwhile, factors such as dietary shifts and short-term environmental changes can disturb and weaken the differences in microbial communities caused by host genetics among closely related host species. The phylosymbiotic signal of oral microbiota, as detected by diverse methods and metrics, appears unstable. This instability may be attributed to the limited number of host species covered for this body site and more than half of the hosts share the same evolutionary divergence time [28].

None of the methods detected a phylosymbiotic signal in the gut (or feces) as strong as that observed in many mammalian species (Fig. 2D; Supplementary Figs S20, S22, S24, S25, S27; Supplementary Tables S11–S24, S26, S27, S29, S30, S32, S33, S35–S37). This may be attributed to the higher substrate availability and anaerobic conditions in the gut, which promote microbial proliferation, potentially increasing the stochasticity of microbial community composition and thereby weakening phylosymbiosis. This finding partly supports prior research that geography and diet influence reptilian gut microbiota more than host genetics (i.e. distantly related species in similar environments or with similar diets show similar gut microbial profiles), corroborated by two recent studies [29–31]. Many studies suggest that phylosymbiotic signals are prevalent in most mammals (although there are a few exceptions) [1, 2, 32], but inconclusive in other vertebrates [2, 6–10], possibly due to unique mammalian traits enhancing host control of microbes, thus promoting phylosymbiosis. These traits include viviparity, lactation, parental care, social behaviors, and adaptive immunity [3]. Herpetofauna are generally not viviparous (with only a minority exhibiting ovoviviparity) and lack the capacity for lactation. Compared to mammals, most of them exhibit less parental care and adaptive immunity. These factors diminish the role of host phylogeny in microbial community assembly. Similar to the gut, none of the methods detected a strong phylosymbiotic signal in herpetofaunal skin (Supplementary Figs S15–S17, S23, S26; Supplementary Tables S11–S22, S31, S34, S35), which supports the viewpoint of Bletz et al. (2017) rather than that of Osborne et al. (2024) [11, 12]. The strong phylosymbiosis signal observed in Osborne et al. (2024) may result from co-variation between host species and habitat (without overlapping ranges and ecological niches) [12]. In contrast, in our study and that of Bletz et al. (2017), different hosts share a same habitat, or one host is dispersed across different habitats [11]. These indicate that external factors may have a more powerful shaping effect on skin microbiota than host phylogeny.

Overall, our results indicate the complexity of herpetofaunal microbial communities’ assembly. The extent to which different ecological processes (such as host selection, organismal dispersion, ecological drift, and diversification) shape the symbiotic microbial communities of herpetofauna may depend on body site. Notably, potential confounding factors (e.g. variations in sampling approaches, primer biases, batch effects, and uneven sample distribution) were unavoidable in this study. These factors may have weakened or enhanced the phylosymbiotic signals to some extent [2]. Future research will be facilitated by more comprehensive host species coverage, more sensitive and stable detection methods [33], and unified experimental methods and evaluation standards [34].

## Supplementary Material

Supplementary_text7_wrag076

Supplementary_Table_S1_wrag076

## Data Availability

The list of accession numbers of the raw data in this study is available in Supplementary Table S1.

## Code availability

Main code is available via GitHub at https://github.com/gaoyuze/symbiotic_microbiota_analysis.
