# Supplementary material for: Unique microbial communities and phylosymbiosis signals in herpetofauna
Source: ISME J. 2026 Apr 2;20(1):wrag076. doi: 10.1093/ismejo/wrag076 (PMC13147448; doi:10.1093/ismejo/wrag076)
Supplement: Supplementary_text7_wrag076 [file supplementary_text7_wrag076.pdf]

Supplementary information for the manuscript "Unique microbial communities and phylosymbiosis signals in herpetofauna"

Jiaying Li[3†*], Yuze Gao[2†], Xuelin Zhao[7†], Jiahao Zhu[2†], Si Zheng[2,5], Qihan Guo[2], Longhui Zhao[8], Guocheng Shu[9], Yuzhou Gong[10,11], Wujie Xu[1*], Ting Chen[2,4,6*]

1. South China Sea Fisheries Research Institute, Chinese Academy of Fishery Sciences, Guangzhou 510300, China;
2. Department of Computer Science and Technology, Tsinghua University, Beijing 100084, China;
3. Department of Automation, Tsinghua University, Beijing 100084, China;
4. State Key Laboratory of Complex, Severe, and Rare Diseases, Peking Union Medical College Hospital, Beijing 100730, China;
5. Institute of Medical Information, Chinese Academy of Medical Sciences and Peking Union Medical College, Beijing 100020, China;
6. Department of Bioinformatics, Fujian Key Laboratory of Medical Bioinformatics, Institute of Precision Medicine, School of Medical Technology and Engineering, Fujian Medical University, Fuzhou 350122, China;
7. School of Marine Sciences, Ningbo University, Ningbo 315211, China;
8. Ministry of Education Key Laboratory for Ecology of Tropical Islands, Key Laboratory of Tropical Animal and Plant Ecology of Hainan Province, College of Life Sciences, Hainan Normal University, Haikou, Hainan 571158, China;
9. Faculty of Agriculture, Forest and Food Engineering, Yibin University, Yibin 644000, China;
10. College of Fisheries, Hunan Agricultural University, Changsha 410128, China;
11. Yuelushan Laboratory, Changsha 410128, China.

† These authors contributed equally to this work: Jiaying Li, Yuze Gao, Xuelin Zhao, and Jiahao Zhu

* Corresponding authors: Jiaying Li, Department of Automation, Tsinghua University, 30 Shuangqing Road, Haidian District, Beijing 100084, China. E-mail: jiaying_lee0501@126.com; Wujie Xu, South China Sea Fisheries Research Institute, Chinese Academy of Fishery Sciences, 231 Xingang Road West, Haizhu District, Guangzhou 510300, China. E-mail: xuwujie@scsfri.ac.cn; Ting Chen, Department of Computer Science and Technology, Tsinghua University: 30 Shuangqing Road, Haidian District, Beijing 100084, China. E-mail: tingchen@tsinghua.edu.cn

1. SUPPLEMENTARY MATERIALS AND METHODS

High–throughput sequencing data collection

The data collection process is as described in our previous study [1]. Briefly, we retrieved *16S rRNA* gene amplicon sequencing data from herpetological microbiota projects through keyword searches in NCBI, GSA, CNGBdb database and relevant literature. The microbial *16S rRNA* gene amplicons must be sequenced using Illumina or 454 pyrosequencing instruments, in accordance with established protocols [2, 3] (https://support.illumina.com). The host taxonomy metadata was meticulously reviewed and verified manually, adhering to the Amphibian Species of the World 6.2 (https://amphibiansoftheworld.amnh.org/), AmphibiaWeb (https://amphibiaweb.org/), THE REPTILE DATABASE (http://reptile–database.org/), Animal Diversity Web (https://animaldiversity.org/), and World Species (https://worldspecies.org/) (Supplementary Table S1). More detailed information is available in the Help Page of our database HMicrodb (https://herpdb.com/Help).

Bioinformatic analysis of microbial high–throughput sequencing data

After undergoing quality control with fastp [4], the standardized pipeline of QIIME 2 (version 2023.7.0) was used for sequencing data analysis (https://docs.qiime2.org/2023.7/index.html) [5]. Briefly, Deblur was employed to denoise all clean data, eliminate chimeras, and generate Amplicon Sequence Variants (ASVs). Following annotation based on Greengenes2 (version 2024.09) [6], singletons and ASVs annotated as mitochondria and chloroplast were removed.

Samples with less than 1000 reads or fewer than 10 ASVs were excluded. We integrated all the independently analyzed projects and performed downstream analyses at the microbial genus level or higher taxonomic levels [7, 8]. To avoid the impact of the divergence in the 16S rRNA gene domains between projects, we grouped samples based on primers (the subset of V4 hypervariables amplified using the 515F and 806R primers covered all body sites, with each body sites subset encompassing multiple host species) and conducted ASV–level comparative analyses only among samples amplified with identical primers. A total of 11,697 samples from 337 host species were retaine, including 7,445 skin samples from 193 species, 79 oral samples from 6 species, 64 stomach sample from 10 species, 2,037 gut samples from 142 species, 1,100 feces samples form 43 species, 203 egg samples from 7 species, 237 tail samples from 1 species, 3 esophagus samples from 1 species, 10 lumina samples and 10 mucosa samples from 1 species, 6 oviduct samples from 1 species, 2 pelvis samples from 1 species.

The diversity (alpha diversity indices: Shannon entropy and Pielou evenness index; beta diversity indices: Bray–Curtis, Jaccard, unweighted and weighted UniFrac metrics) of microbial community were calculated using QIIME 2 plug *diversity* [5]. Then, functional predictions of microbial communities were performed using PICRUST 2 (version 2.5.2) [9], and the dissimilarity of samples were calculated based on the Bray–Curtis distance. The microbial diversity and function were summarized based on the body site and taxonomy of host species.

Variations in alpha diversity between groups were assessed by the Wilcoxon and Kruskal–Wallis test using the QIIME 2 plug *diversity* [5]. Pearson's correlation based on the average value of relative abundance of microbiota at genus level among each body sites was calculated using *cor()* function of R software (version 4.0.3) [10]. PERMANOVA tests were conducted to evaluate the effects of body site and host species on the microbial communities and potential functions using R package *vegan* [11]. To mitigate the potential confounding effect of variations in the number of host species across different body sites when assessing the effects of host species on the beta diversity of microbial communities, we standardized the effective size by calculating partial Omega–squared (parOmega Sq) using R package MicEco (Jakob Russel. (2021). Russel88/MicEco: v0.9.15 (v0.9.15). Zenodo. https://doi.org/10.5281/zenodo.4733747).

Phylosymbiosis analysis

We identified phylosymbiosis using both matrix–based, topology–based and multivariate Brownian motion model–based methods [12, 13]. The host phylogenetic trees were constructed using Timetree (http://timetree.org)[14]. A total of 267 host species had both host phylogeny information and microbiome data available. The microbiota composition of each host species was calculated by averaging the samples per species as previous studies [15]. The beta diversity distance matrices were calculate using QIIME 2 plug *diversity* followed by UPGMA clustered (Unweighted Pair Group Method with Arithmetic Mean, also known as average linkage) [5]. The

correlation between host phylogeny and microbial community was examined using Mantel test based on Pearson's and Spearman's correlations by R package *vegan* [11]. We vectorized host and microbiota matrices, and employed linear regressions to test the effect of host phylogenetic distance on microbiome dissimilarity using *lm()* function in R software as previous studies [10, 15, 16]. To mitigate the impact of differences in sample sizes from different body sites, randomisation procedures were employed prior to conducting the Mantel tests. In brief, we resampled all host species at each body site to reach a uniform target size (this size was determined by the body site with the fewest host species, which in this study was the oral cavity), and calculated the correlation using sub–matrices (999 random permutations were conducted). Ten thousand random samplings were performed on each body site dataset. The Kruskal–Wallis (R package *vegan*) test and the Dunn test (R package *FSA*) were then used to analyse the differences in correlation coefficients obtained through the aforementioned process across different body sites [11, 17].

In terms of the topological comparison method, the Robinson–Foulds (RF) and Matching Cluster (MC) scores were calculated in order to quantify the congruence between the host phylogeny and the microbiota dendrogram (https://github.com/awbrooks19/topological_congruence?files=1) [12, 18]. The RF metric quantifies topological differences between two phylogenetic trees via discordant split counting, whereas the MC metric assesses their overall similarity through identification of the maximum shared cluster set [19, 20]. These RF and MC scores were then normalized to generate the normalized RF (nRF) and normalized

MC (nMC) scores, which represent inconsistency percentages ranging from 0.0 (complete congruence, 0% inconsistency) to 1.0 (complete incongruence, 100% inconsistency) [18]. The significance of the RF and MC analyses was assessed by calculating the probability that randomized bifurcating dendrogram topologies would yield phylosymbiotic patterns as or more congruent than those of the microbiota dendrogram [18].

For body sites (skin, gut, feces) with more than 25 host species, we further identified phylosymbiosis using the multivariate Brownian motion model (ABDOMEN)[13], with 3000 STAN iterations permutated 100 times [21]. Pagel's lambda estimates close to 1 indicate that an untransformed tree explains the data well, reflecting strong phylosymbiosis, whereas values near 0 suggest weak or no explanatory power of the tree and thus minimal or absent phylosymbiosis [22].

Comparison of the symbiotic microbiota among fish, herpetofauna, and mammals

For comparison with the symbiotic microbiota data of fish and mammals, we obtained the raw data from NCBI Sequence Read Archive (SRA) after bibliographic retrieval (Supplementary Table S1). Given that gut microbiota have garnered widespread attention across fish, reptiles, and mammals, we focused on this body site. All these microbiota data were processed by the same pipline as herpetofaunal microbial data (https://github.com/gaoyuze/symbiotic_microbiota_analysis). The main microbiota at the phylum level were compared among different hosts using the Kruskal–Wallis test

through R package *vegan* [11]. Benjamini–Hochberg False Discovery Rate (FDR) adjusted P value of ≤ 0.05. All parameters are selected with default values except for special declarations.

2. SUPPLEMENTARY RESULTS

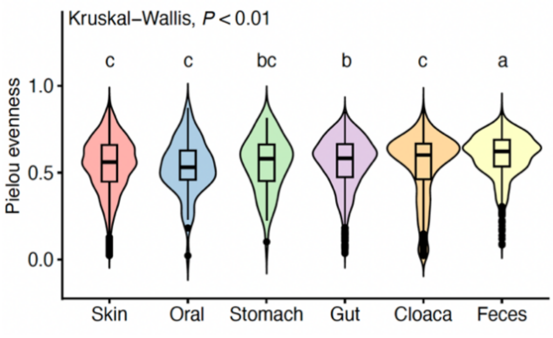

Supplementary Fig. S1 | Alpha diversity of Pielou evenness index at genus level in samples grouped by host body sites. Different letters above box plots indicate significant differences between body sites, respectively ($P < 0.05$).

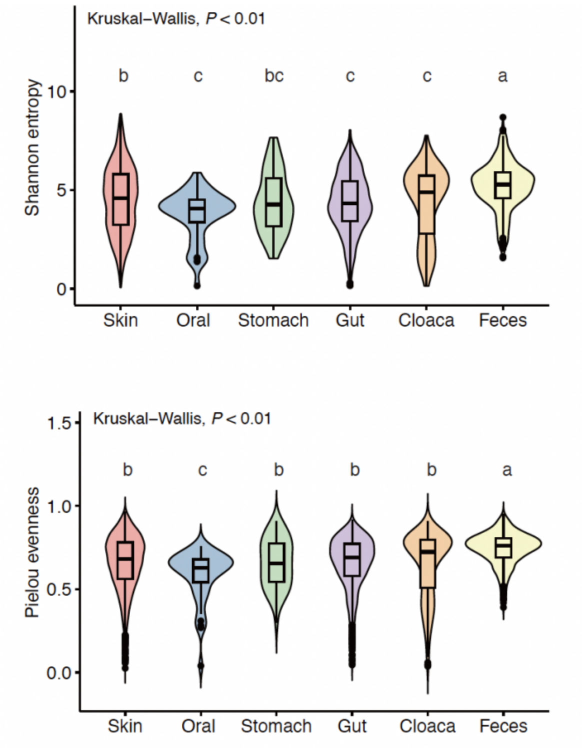

Supplementary Fig. S2 | Alpha diversity of Shannon entropy and Pielou evenness index at ASV level in samples grouped by host body sites. Different letters above box plots indicate significant differences between body sites, respectively ($P < 0.05$).

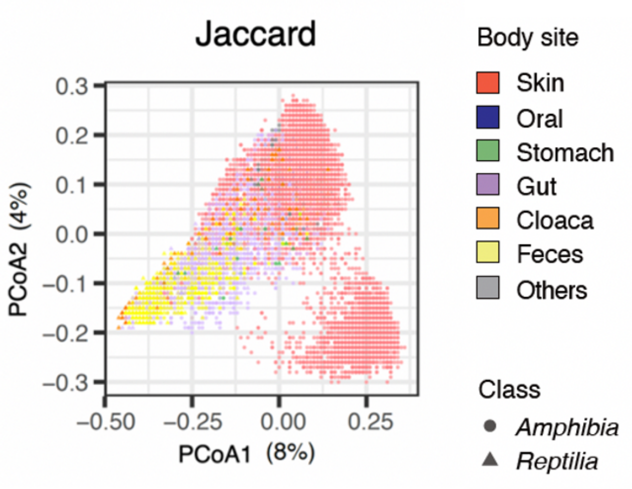

Supplementary Fig. S3 | Beta diversity measured by principal coordinates analysis (PCoA) based on Jaccard distance for microbial community at genus level. Each point was colored by the corresponding sample belonged host body site and shaped by host class. The category "Others" includes esophagus, oviduct, pelvis, lumina, mucosa, egg, and tail. The outliers were not shown. PERMANOVA results for the effects of body site, host species and their interaction are shown in Supplementary Table S3.

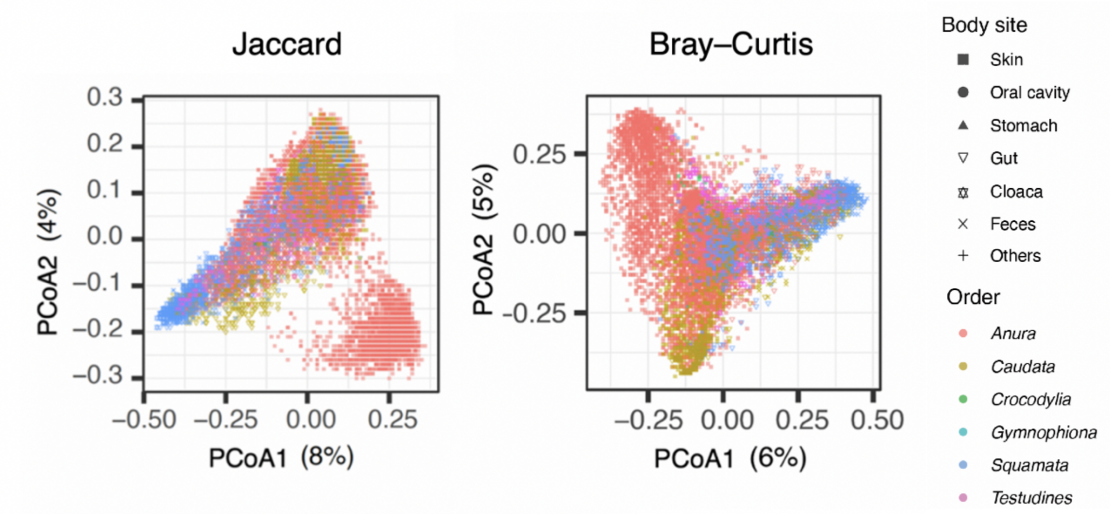

Supplementary Fig. S4 | Beta diversity measured by PCoA based on Jaccard (left) and Bray–Curtis (right) distance for microbial community at genus level. Each point was shaped by host body site and colored by the corresponding sample belonged host order. The outliers were not shown. PERMANOVA results for the effects of body site, host species and their interaction are shown in Supplementary Table S4.

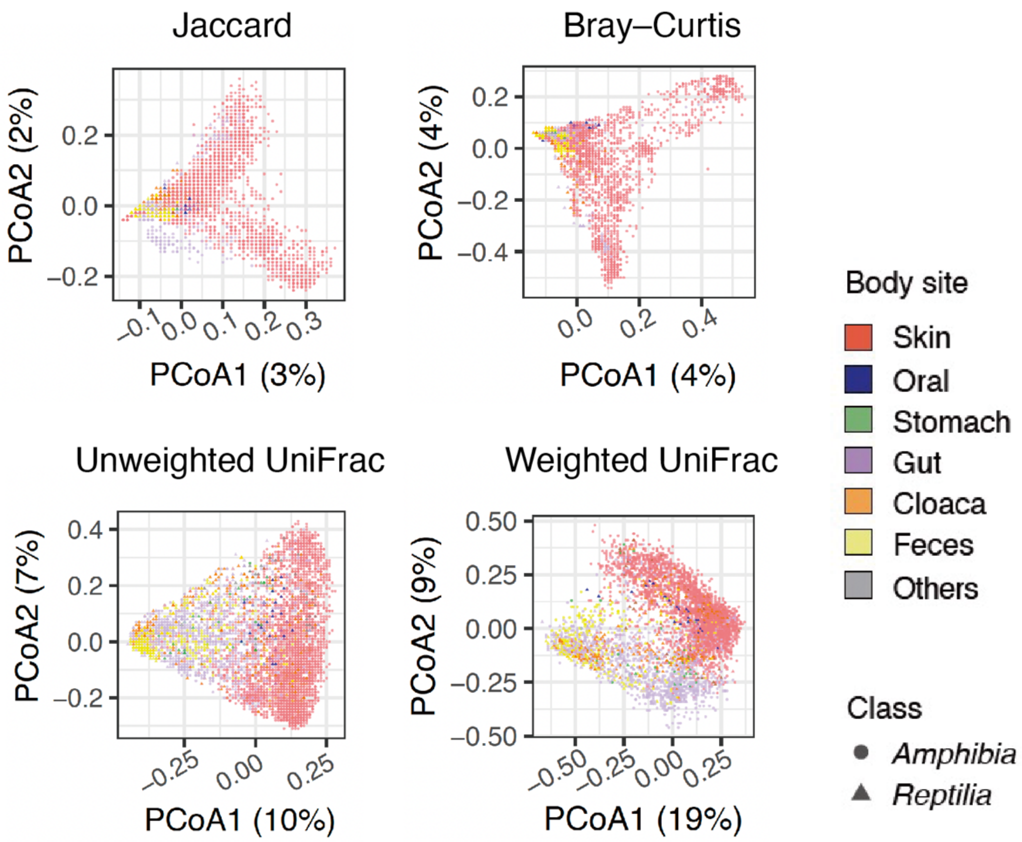

Supplementary Fig. S5 | Beta diversity measured by PCoA based on Jaccard, Bray–Curtis, unweighted UniFrac and weighted UniFrac distance for microbial community at ASV level. Each point was colored by the corresponding sample belonged host body site and shaped by host class. The outliers were not shown. PERMANOVA results for the effects of body site, host species and their interaction are shown in Supplementary Table S5–S8.

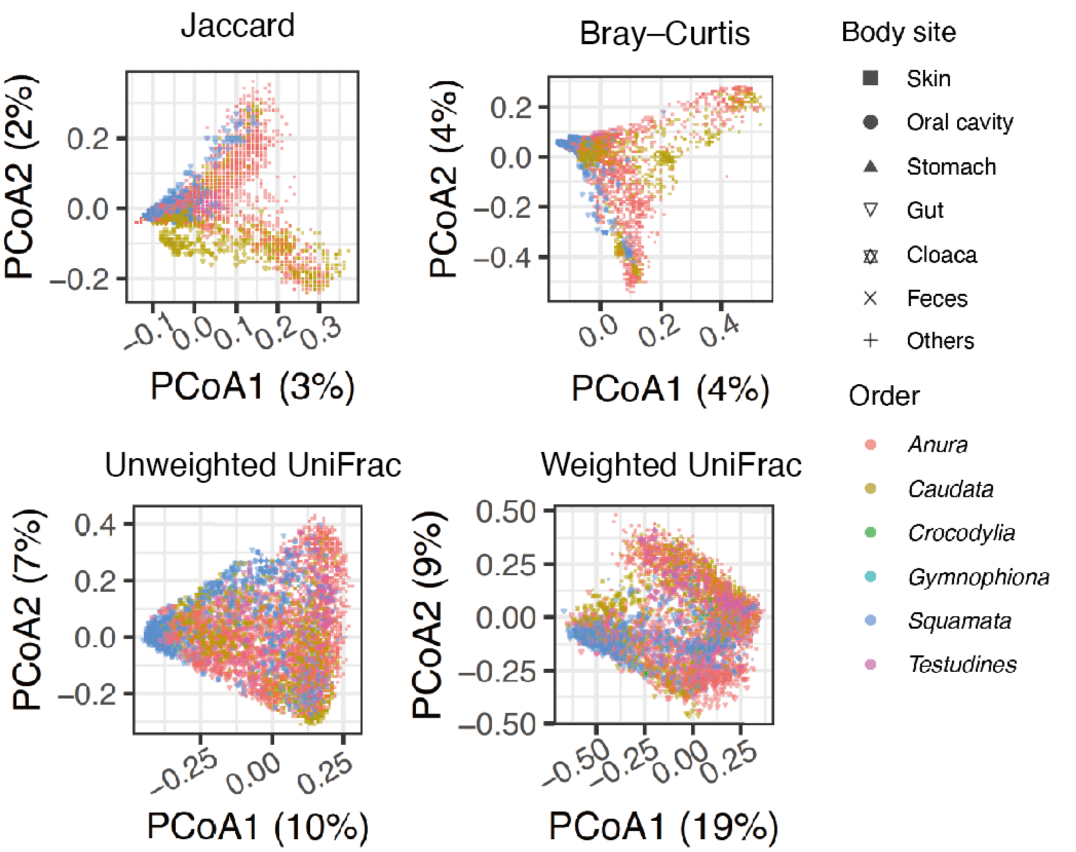

Supplementary Fig. S6 | Beta diversity measured by PCoA based on Jaccard, Bray–Curtis, unweighted UniFrac and weighted UniFrac distance for microbial community at ASV level. Each point was colored by the corresponding sample belonged host order and shaped by host body site. The outliers were not shown. PERMANOVA results for the effects of body site, host species and their interaction are shown in Supplementary Table S5–S8.

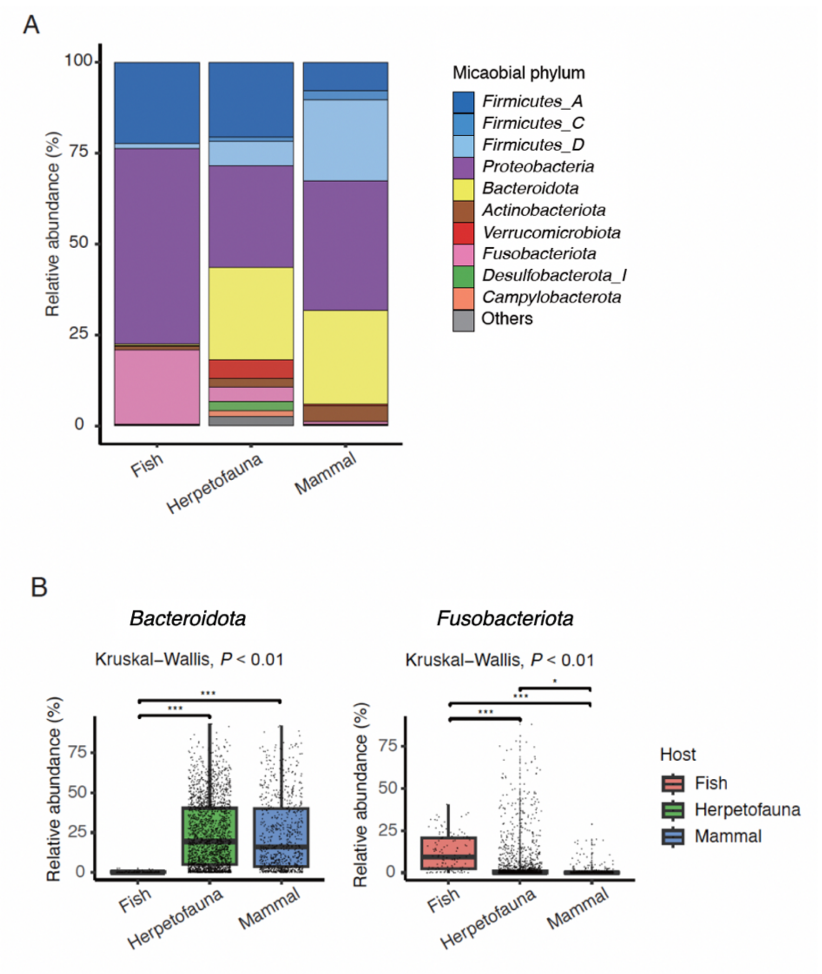

Supplementary Fig. S7 | Comparative analysis of gut microbiota across fish, herpetofauna, and mammal. **A,** Relative abundances of the major microbial phyla across fish, herpetofauna, and mammal. **B,** Relative abundance of Bacteroidota and Fusobacteriota across fish, herpetofauna, and mammal.

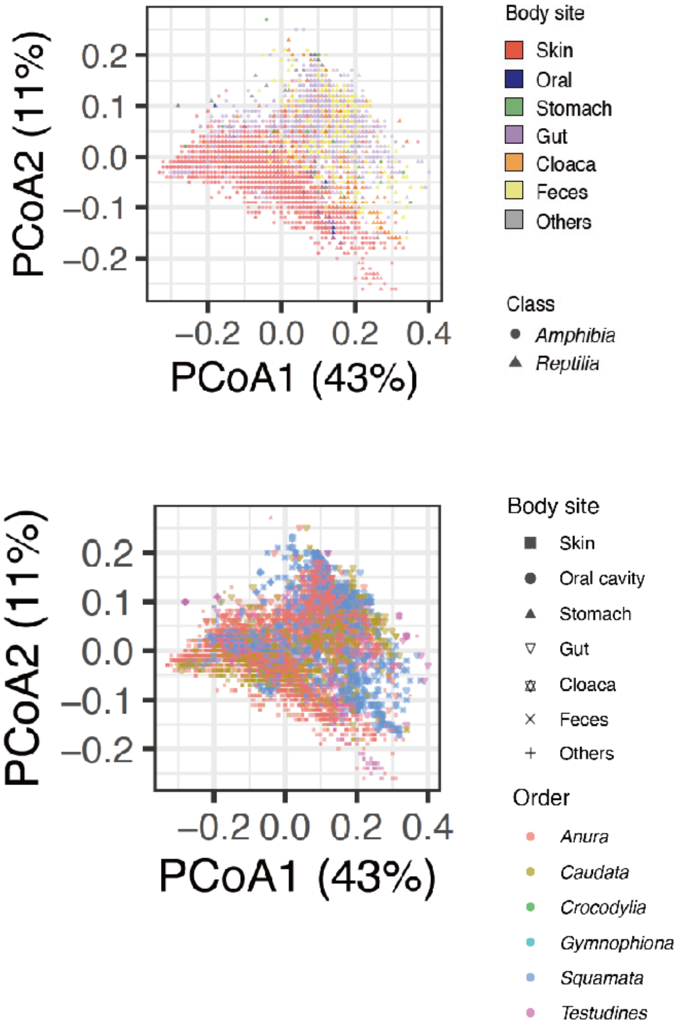

Supplementary Fig. S8 | **A,** PCoA of samples based on Bray–Curtis dissimilarity for the microbial functions. Each point was colored by the corresponding sample belonged host body site and shaped by host class (top) or colored by the corresponding sample belonged host order and shaped by host body site (bottom). PERMANOVA results for the effects of body site, host species and their interaction are shown in Supplementary Table S9.

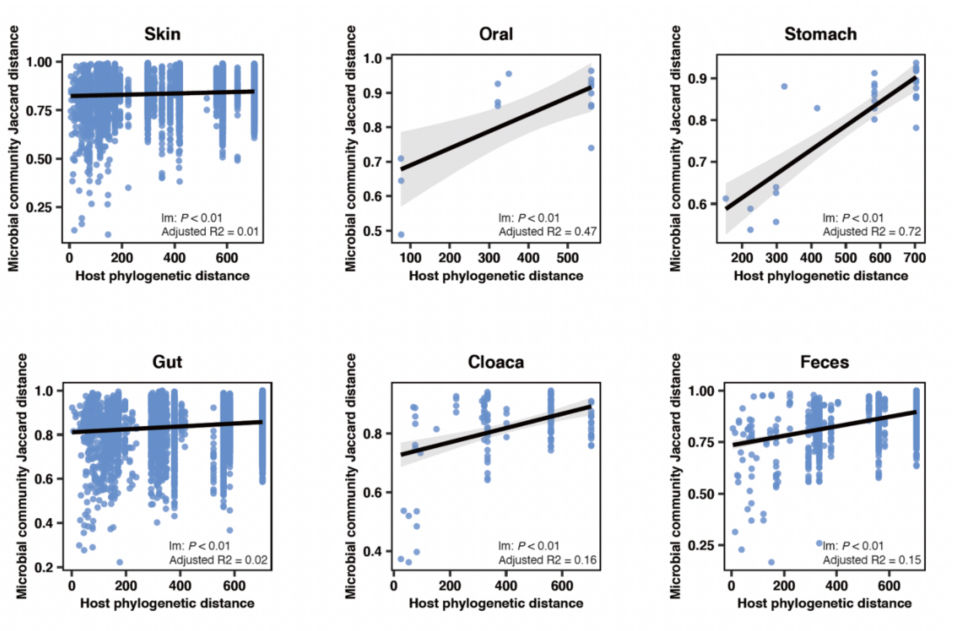

Supplementary Fig. S9 | Pairwise comparison between host phylogenetic distance and the Jaccard distance of their symbiotic microbiota at genus level. The lines represent the trend lines created using linear models, and the gray shadows indicate 95% confidence intervals. Mantel tests based on Pearson's and Spearman's correlation are shown in Supplementary Table S11.

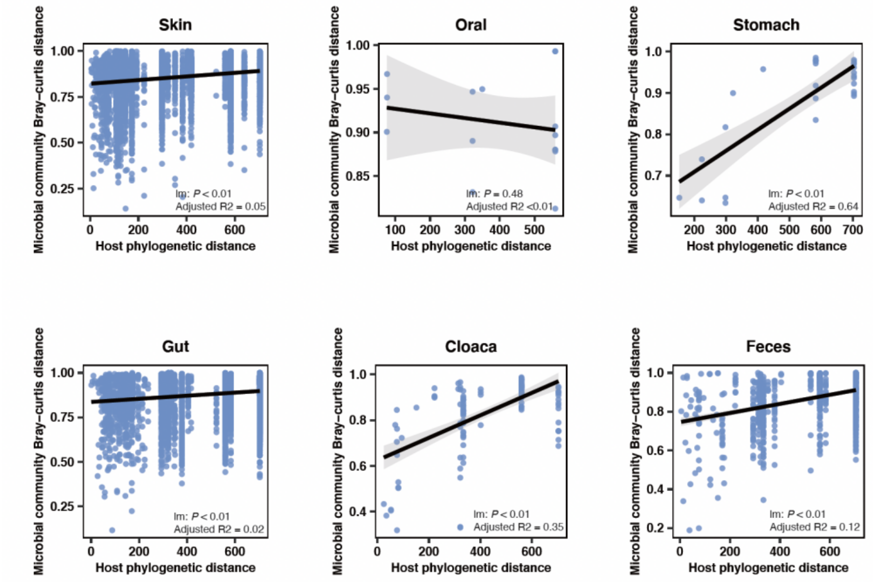

Supplementary Fig. S10 | Pairwise comparison between host phylogenetic distance and the Bray–Curtis distance of their symbiotic microbiota at genus level. The lines represent the trend lines created using linear models, and the gray shadows indicate 95% confidence intervals. Mantel tests based on Pearson's and Spearman's correlation are shown in Supplementary Table S12.

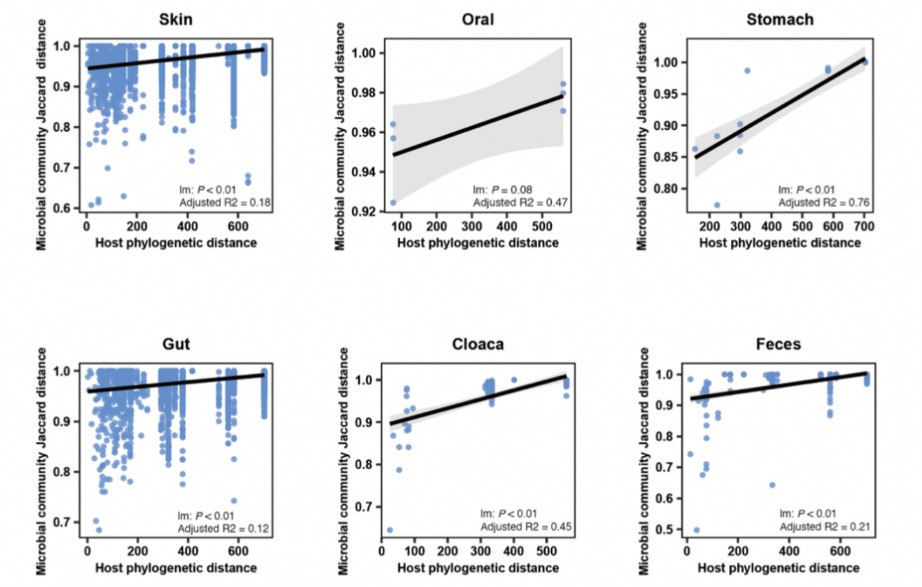

Supplementary Fig. S11 | Pairwise comparison between host phylogenetic distance and the Jaccard distance of their symbiotic microbiota at ASV level. The lines represent the trend lines created using linear models, and the gray shadows indicate 95% confidence intervals. Mantel tests based on Pearson’s and Spearman’s correlation are shown in Supplementary Table S13.

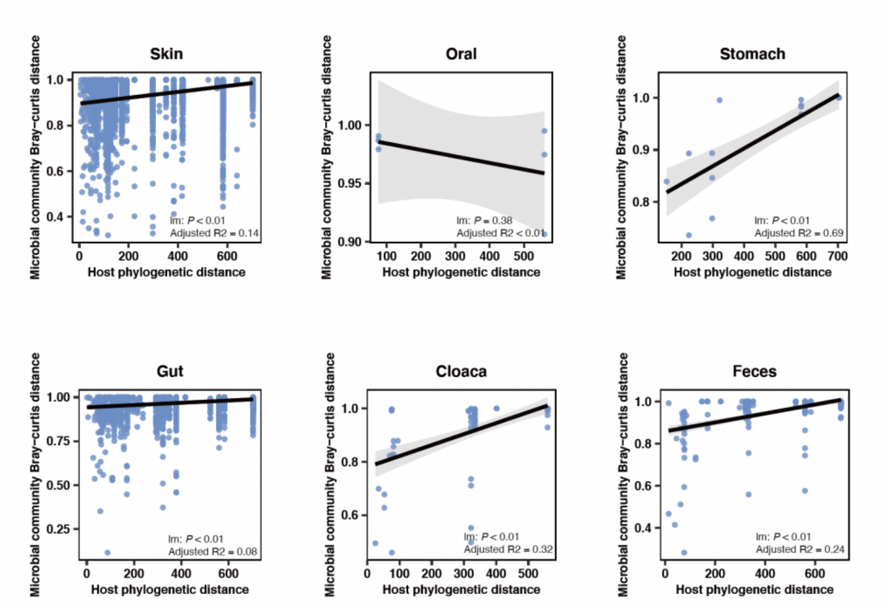

Supplementary Fig. S12 | Pairwise comparison between host phylogenetic distance and the Bray–Curtis distance of their symbiotic microbiota at ASV level. The lines represent the trend lines created using linear models, and the gray shadows indicate 95% confidence intervals. Mantel tests based on Pearson’s and Spearman’s correlation are shown in Supplementary Table S14.

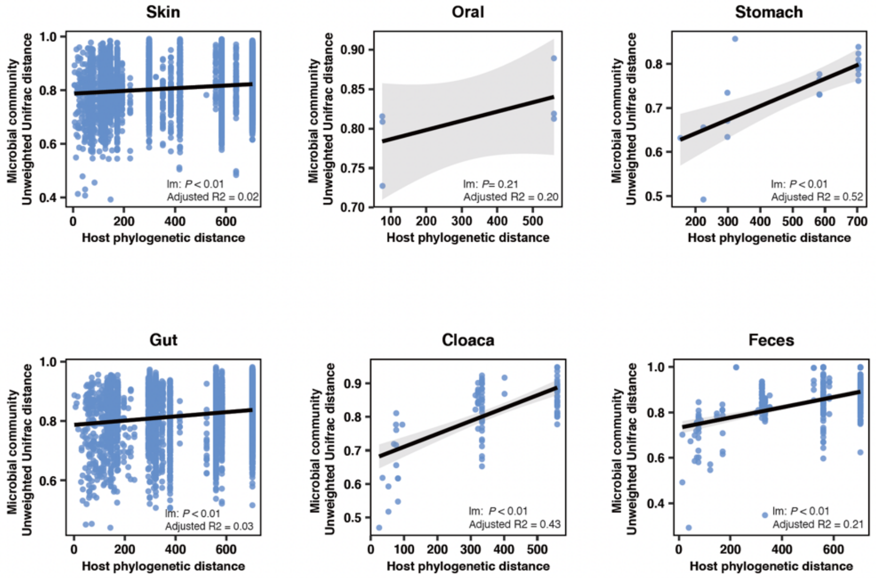

Supplementary Fig. S13 | Pairwise comparison between host phylogenetic distance and the unweighted UniFrac distance of their symbiotic microbiota at ASV level. The lines represent the trend lines created using linear models, and the gray shadows indicate 95% confidence intervals. Mantel tests based on Pearson's and Spearman's correlation are shown in Supplementary Table S15.

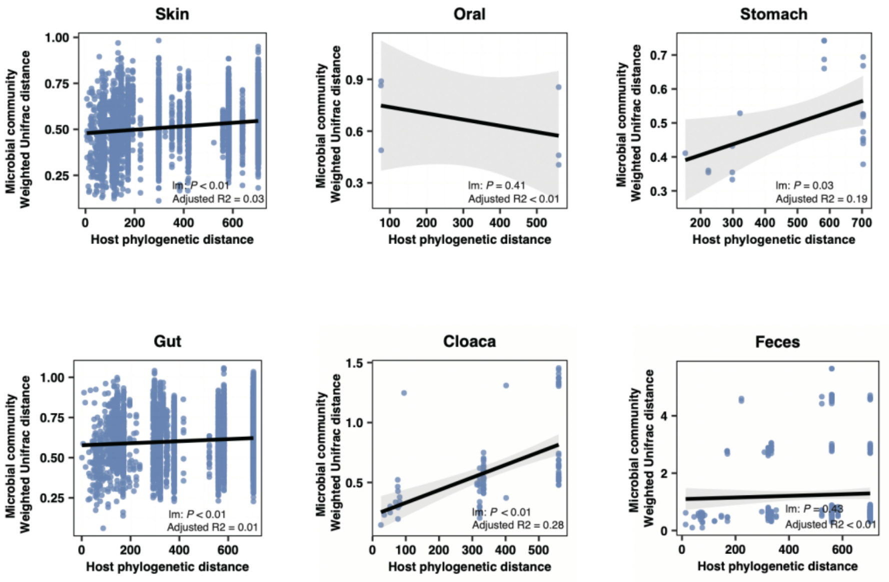

Supplementary Fig. S14 | Pairwise comparison between host phylogenetic distance and the weighted UniFrac distance of their symbiotic microbiota at ASV level. The lines represent the trend lines created using linear models, and the gray shadows indicate 95% confidence intervals. Mantel tests based on Pearson's and Spearman's correlation are shown in Supplementary Table S16.

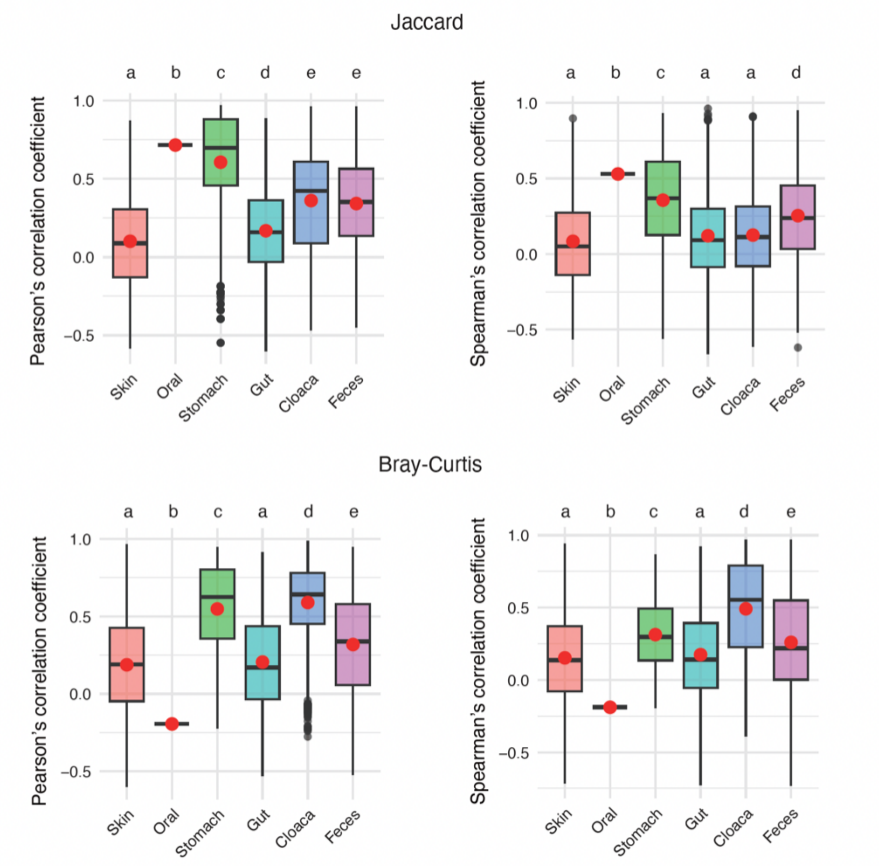

Supplementary Fig. S15 | The distribution of the Pearson's (left) and Spearman's (right) correlation coefficient between the phylogenetic distance of the host and the beta diversity distance based on Jaccard and Bray–Curtis metrics of the microbial communities at genus level. Host species at each body site were resampled to a uniform size (determined by the oral cavity, the site with fewest species). Correlations were calculated via sub–matrices (999 permutations), with 10,000 random samplings per body site dataset. The red point marks the mean correlation of each body site. Thicker dots represent outlier values. Different letters above box plots indicate significant differences between body sites, respectively ($P < 0.05$).

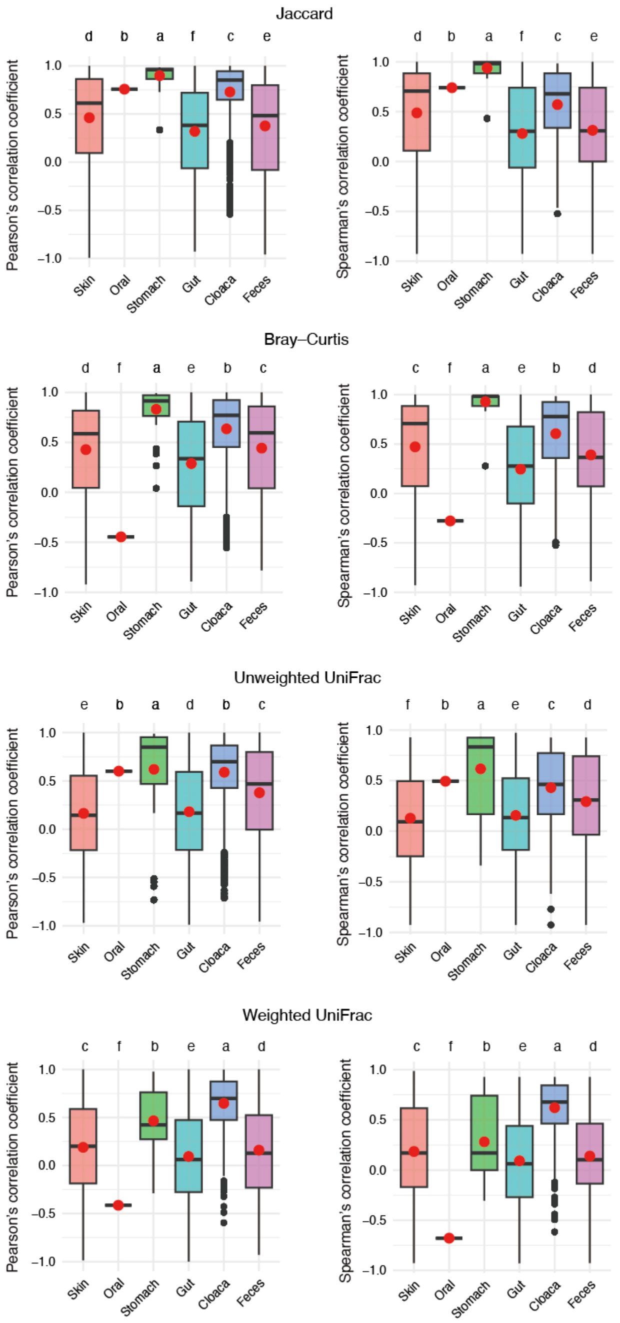

Supplementary Fig. S16 | The distribution of the Pearson's (left) and Spearman's (right) correlation coefficient between the phylogenetic distance of the host and the beta diversity distance based on Jaccard, Bray–Curtis, unweighted UniFrac, and weighted UniFrac distance metrics of the microbial communities at ASV level. Host species at each body site were resampled to a uniform size (determined by the oral cavity, the site with fewest species). Correlations were calculated via sub–matrices (999 permutations), with 10,000 random samplings per body site dataset. The red point marks the mean correlation of each body site. Thicker dots represent outlier values. Different letters above box plots indicate significant differences between body sites, respectively (P< 0.05).

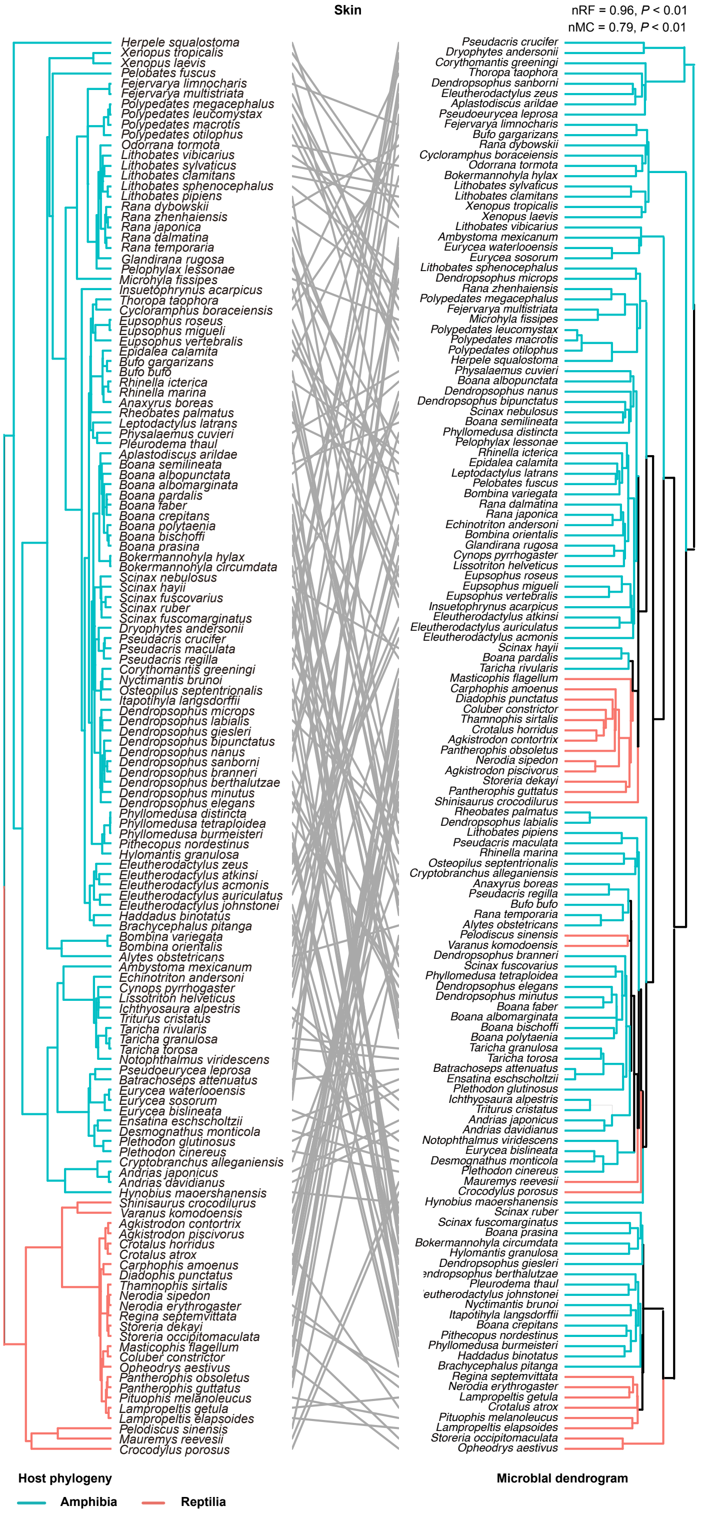

Supplementary Fig. S17 | The host species phylogenetic tree (left) and the skin microbiota dendrogram based on Jaccard distance metrics at genus level (right). Nomalized Robinson–Foulds (nRF) and Nomalized Matching Cluster (nMC) scale from 0.0 (complete congruence) to 1.0 (complete incongruence).

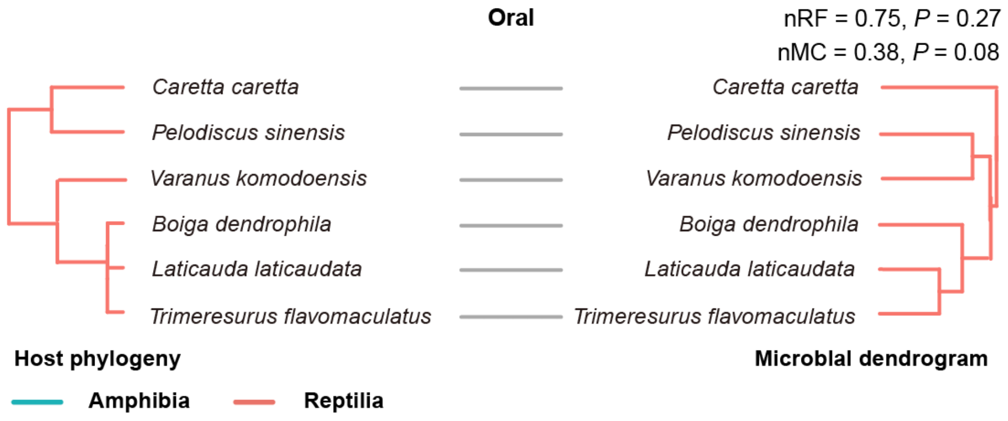

Supplementary Fig. S18 | The host species phylogenetic tree (left) and the oral microbiota dendrogram based on Jaccard distance metrics at genus level (right). Nomalized Robinson–Foulds (nRF) and Nomalized Matching Cluster (nMC) scale from 0.0 (complete congruence) to 1.0 (complete incongruence).

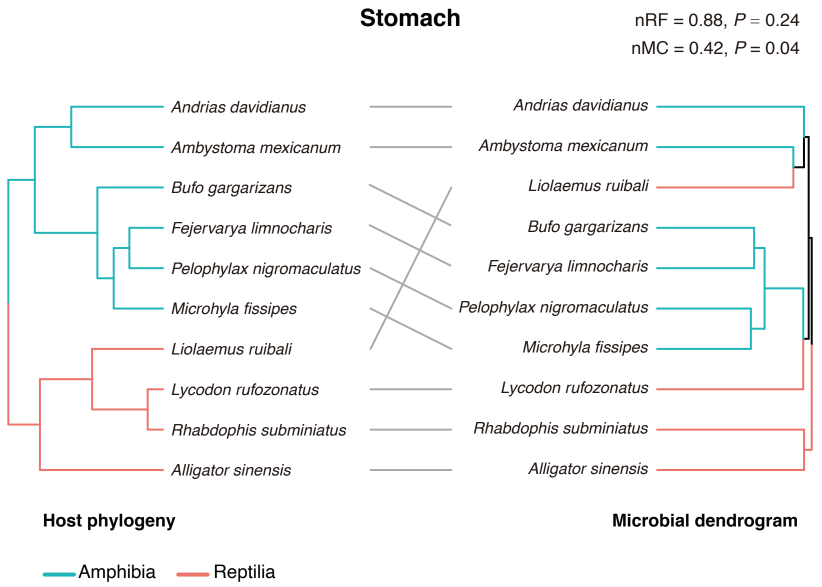

Supplementary Fig. S19 | The host species phylogenetic tree (left) and the stomach microbiota dendrogram based on Jaccard distance metrics at genus level (right). Nomalized Robinson–Foulds (nRF) and Nomalized Matching Cluster (nMC) scale from 0.0 (complete congruence) to 1.0 (complete incongruence).

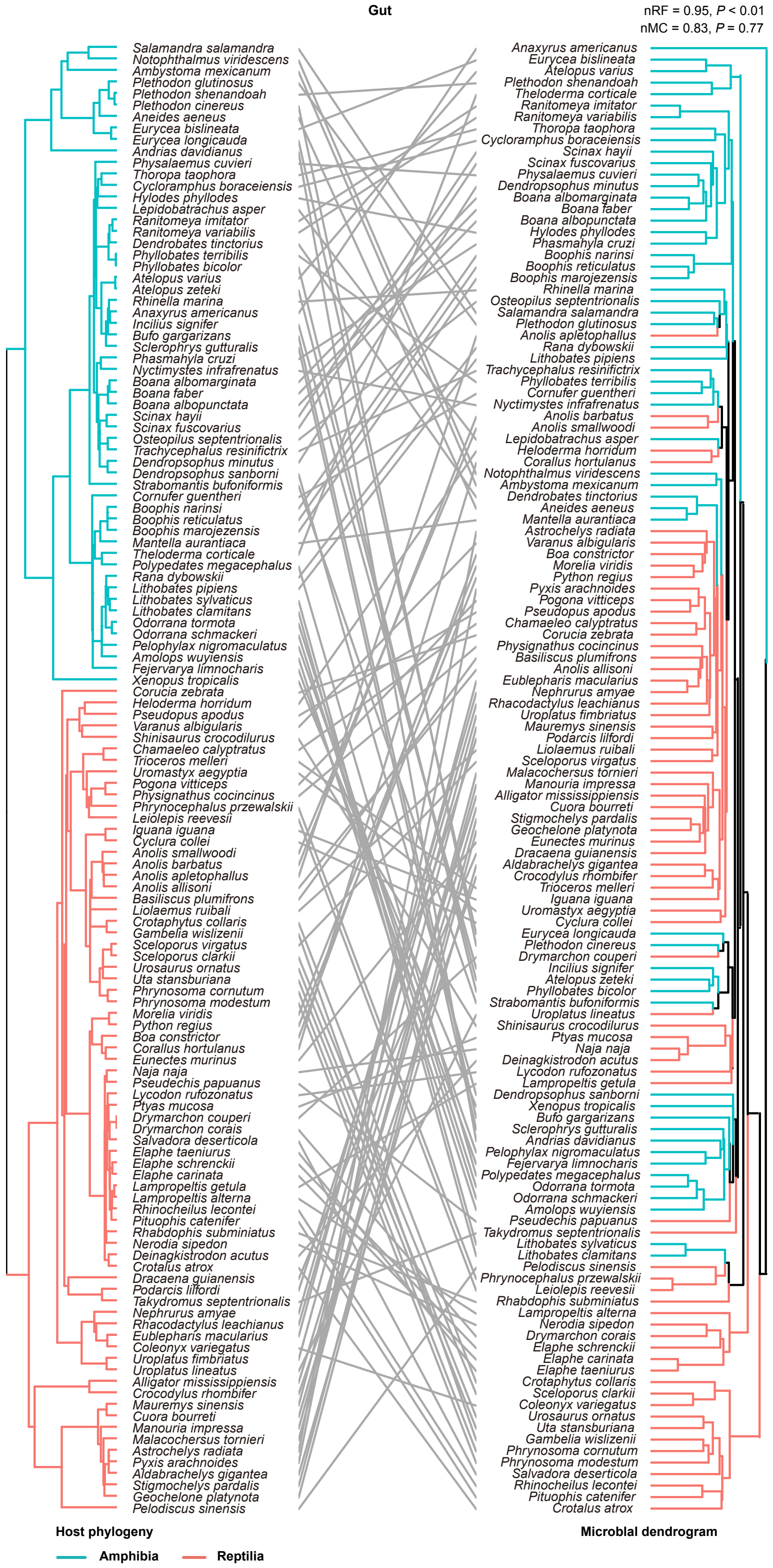

Supplementary Fig. S20 | The host species phylogenetic tree (left) and the gut microbiota dendrogram based on Jaccard distance metrics at genus level (right). Nomalized Robinson–Foulds (nRF) and Nomalized Matching Cluster (nMC) scale from 0.0 (complete congruence) to 1.0 (complete incongruence).

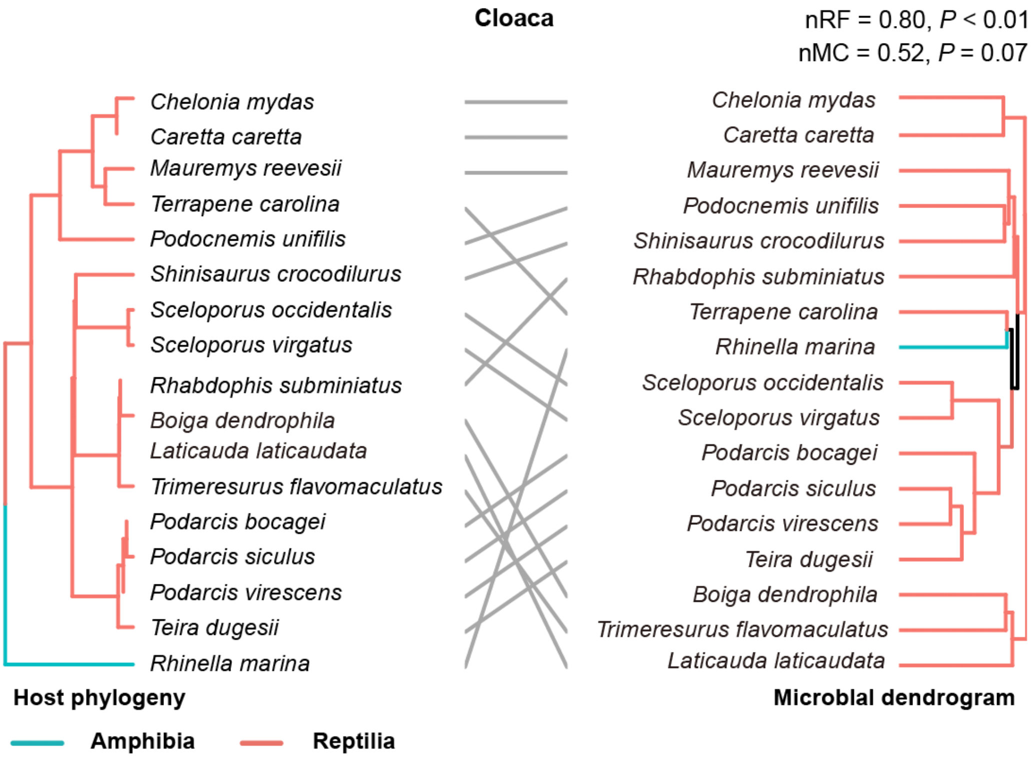

Supplementary Fig. S21 | The host species phylogenetic tree (left) and the cloaca microbiota dendrogram based on Jaccard distance metrics at genus level (right). Nomalized Robinson–Foulds (nRF) and Nomalized Matching Cluster (nMC) scale from 0.0 (complete congruence) to 1.0 (complete incongruence).

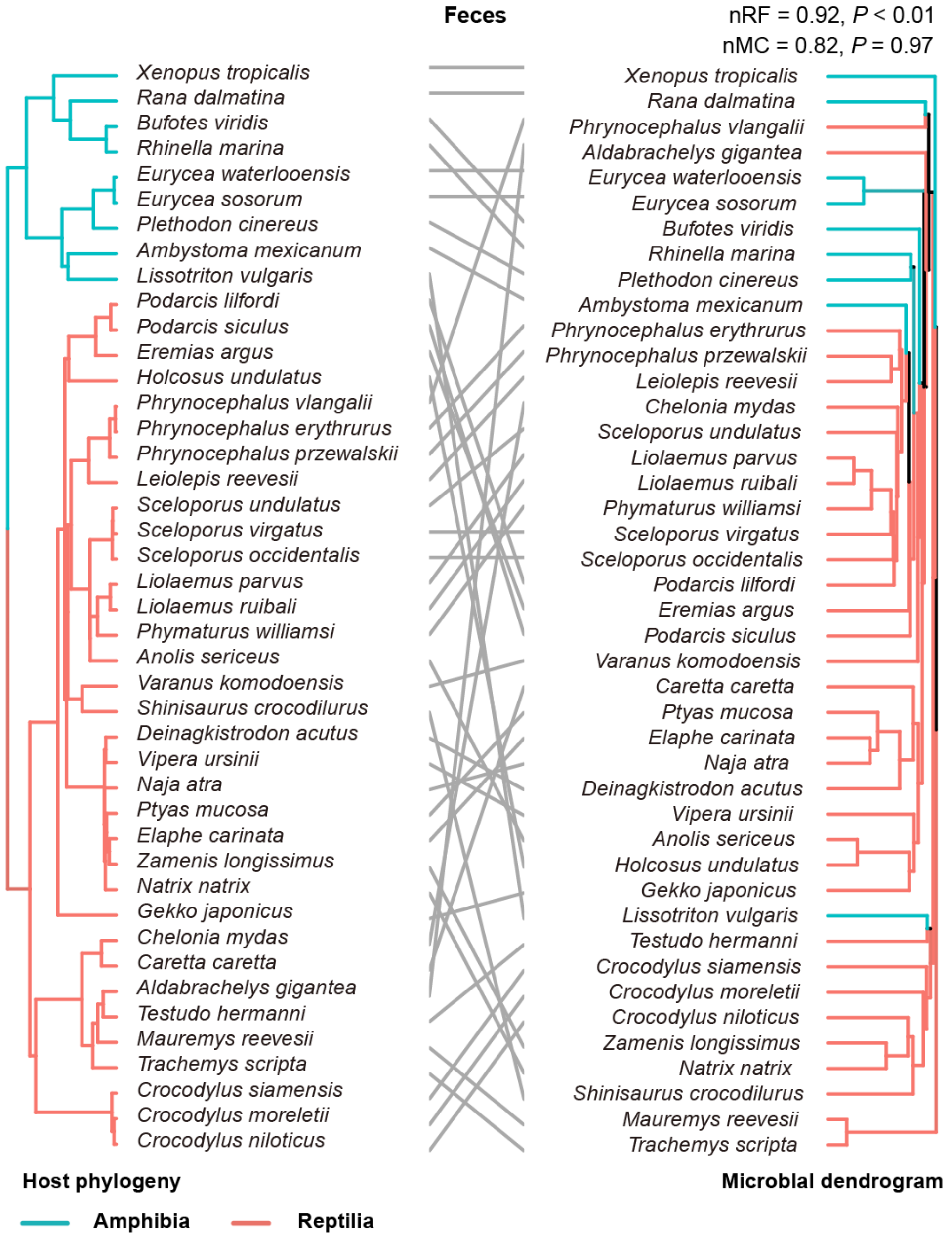

Supplementary Fig. S22 | The host species phylogenetic tree (left) and the feces microbiota dendrogram based on Jaccard distance metrics at genus level (right). Nomalized Robinson–Foulds (nRF) and Nomalized Matching Cluster (nMC) scale from 0.0 (complete congruence) to 1.0 (complete incongruence).

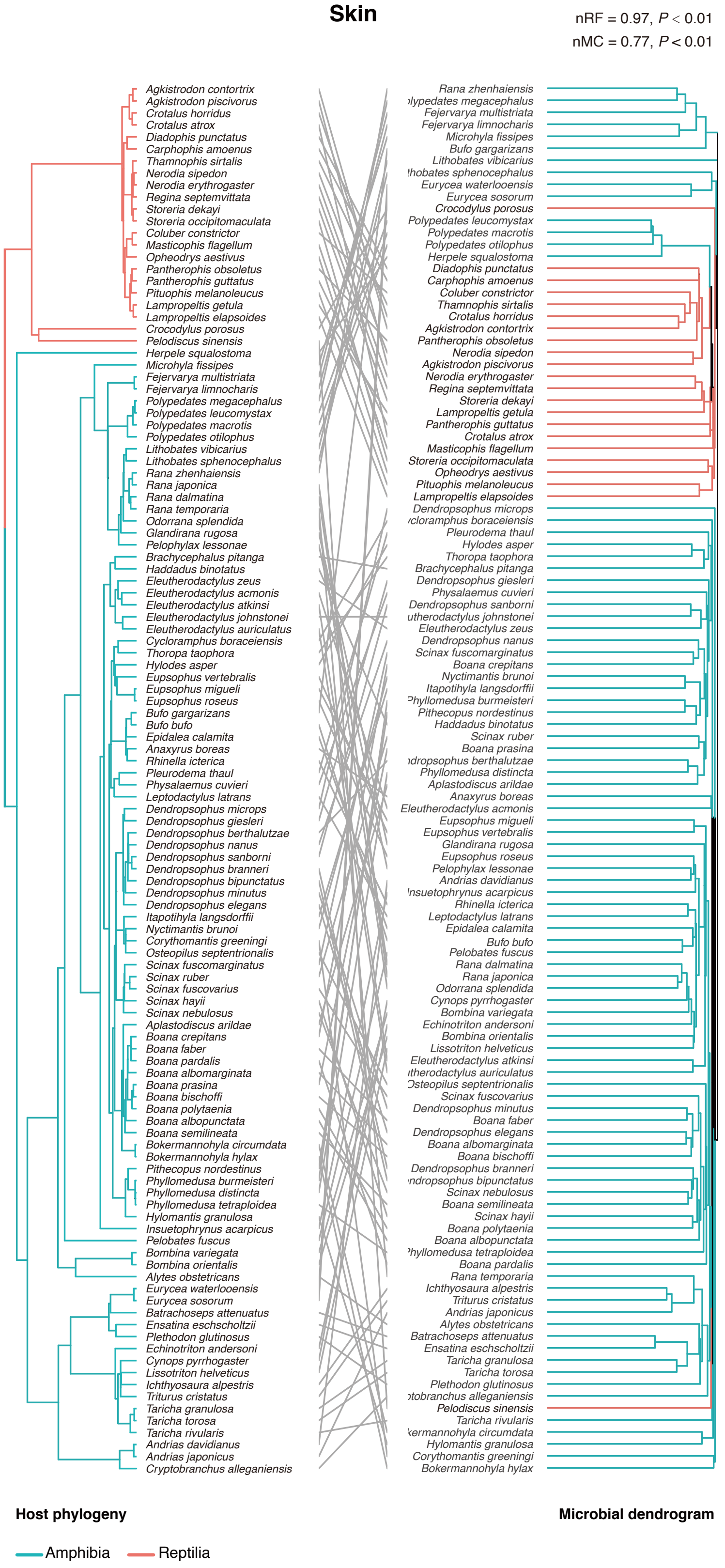

Supplementary Fig. S23 | The host species phylogenetic tree (left) and the skin microbiota dendrogram based on Jaccard distance metrics at ASV level (right). Nomalized Robinson–Foulds (nRF) and Nomalized Matching Cluster (nMC) scale from 0.0 (complete congruence) to 1.0 (complete incongruence).

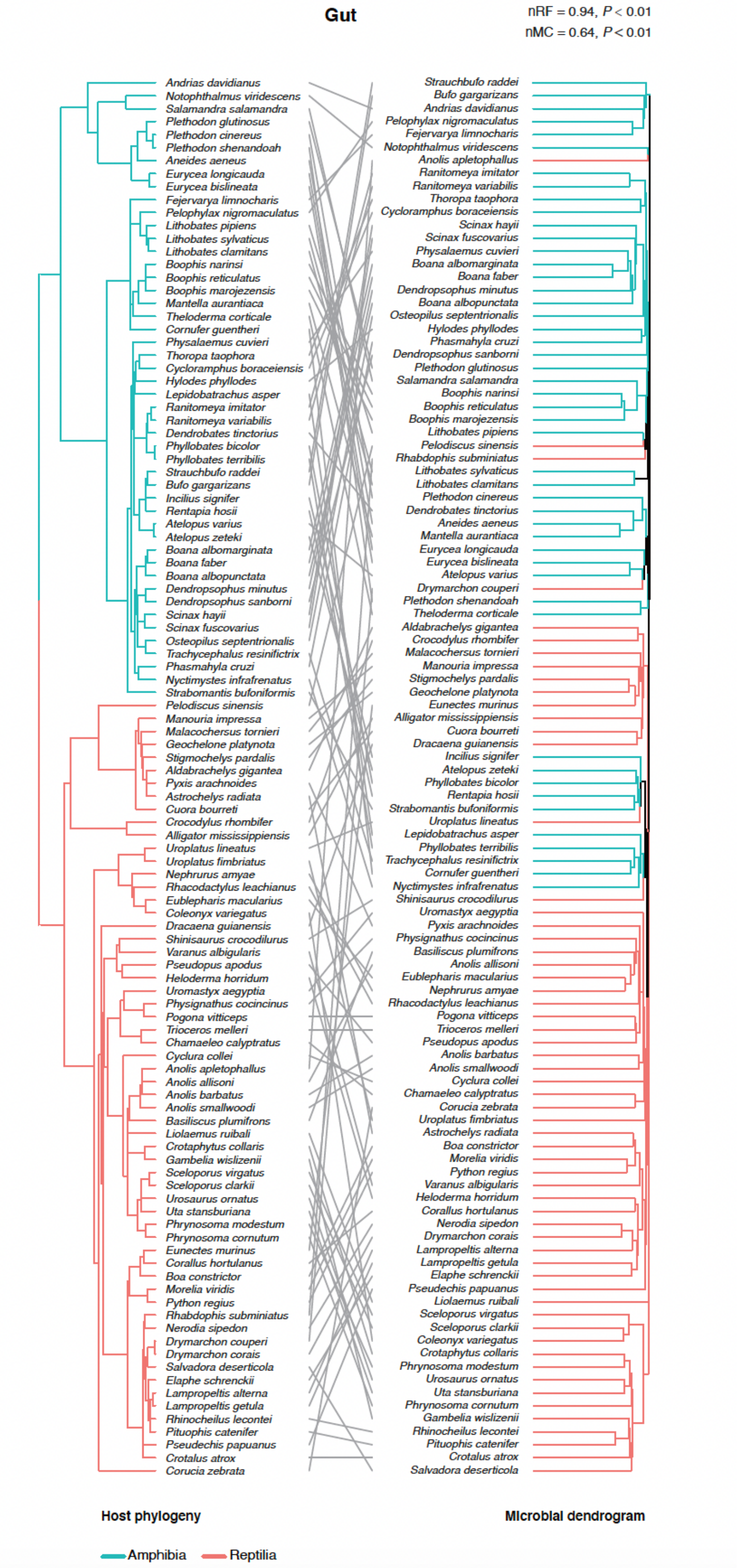

Supplementary Fig. S24 | The host species phylogenetic tree (left) and the gut microbiota dendrogram based on Jaccard distance metrics at ASV level (right). Nomalized Robinson–Foulds (nRF) and Nomalized Matching Cluster (nMC) scale from 0.0 (complete congruence) to 1.0 (complete incongruence).

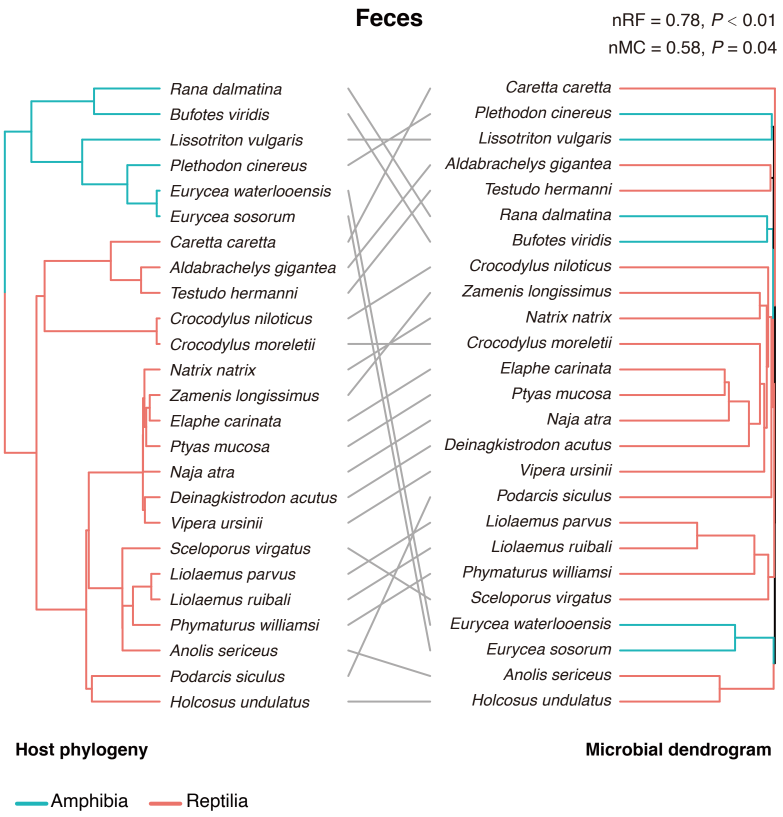

Supplementary Fig. S25 | The host species phylogenetic tree (left) and the feces microbiota dendrogram based on Jaccard distance metrics at ASV level (right). Nomalized Robinson–Foulds (nRF) and Nomalized Matching Cluster (nMC) scale from 0.0 (complete congruence) to 1.0 (complete incongruence).

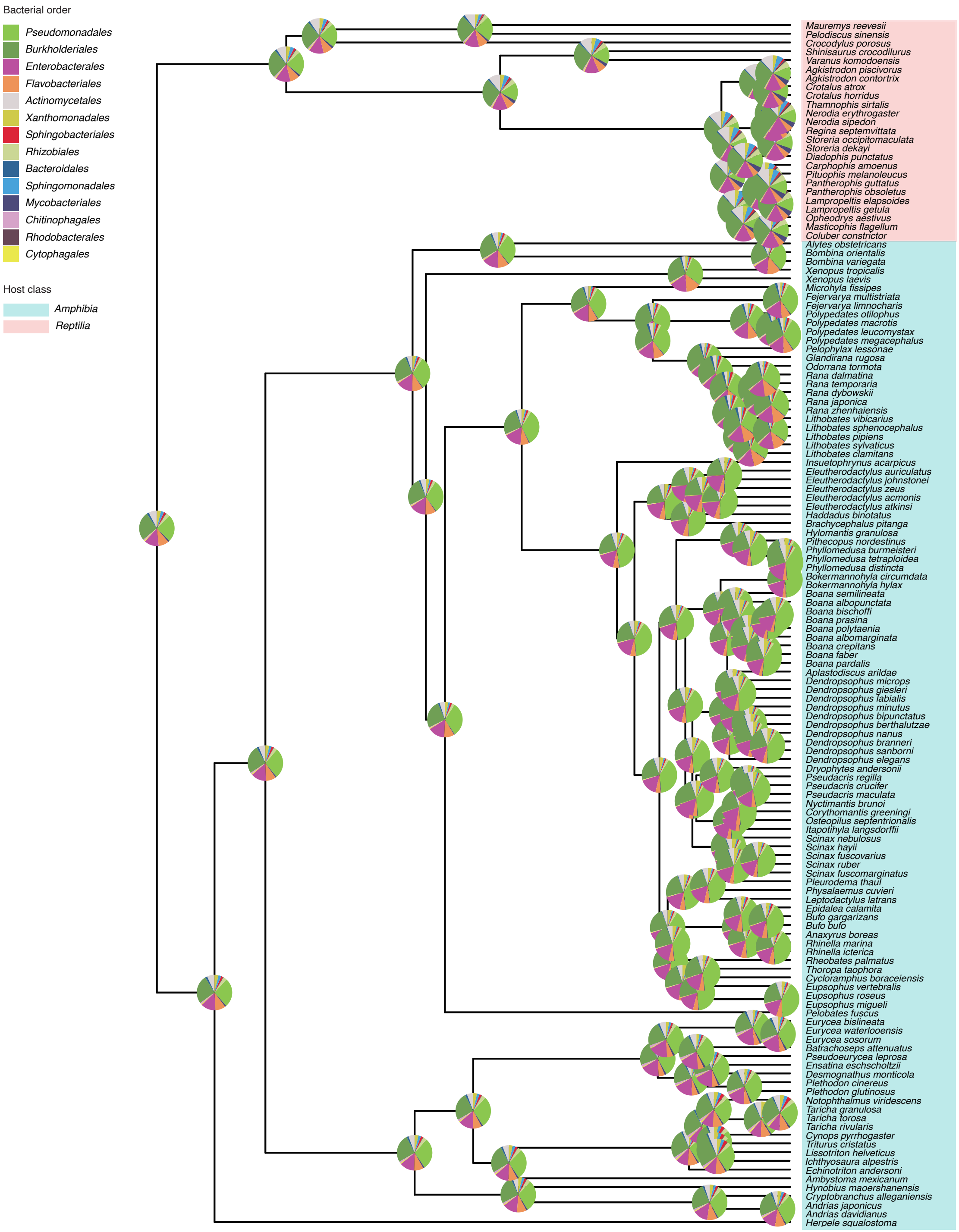

Supplementary Fig. S26 | Ancestral reconstruction of skin microbiota with phylogenetic tree of the host species and associated relative abundances of the 14 most abundant bacterial orders. The pie charts at the root and nodes of the tree show the estimated ancestral microbiota compositions (the mean of the posterior distribution at the root, and the generalized least squares estimates at the other internal nodes).

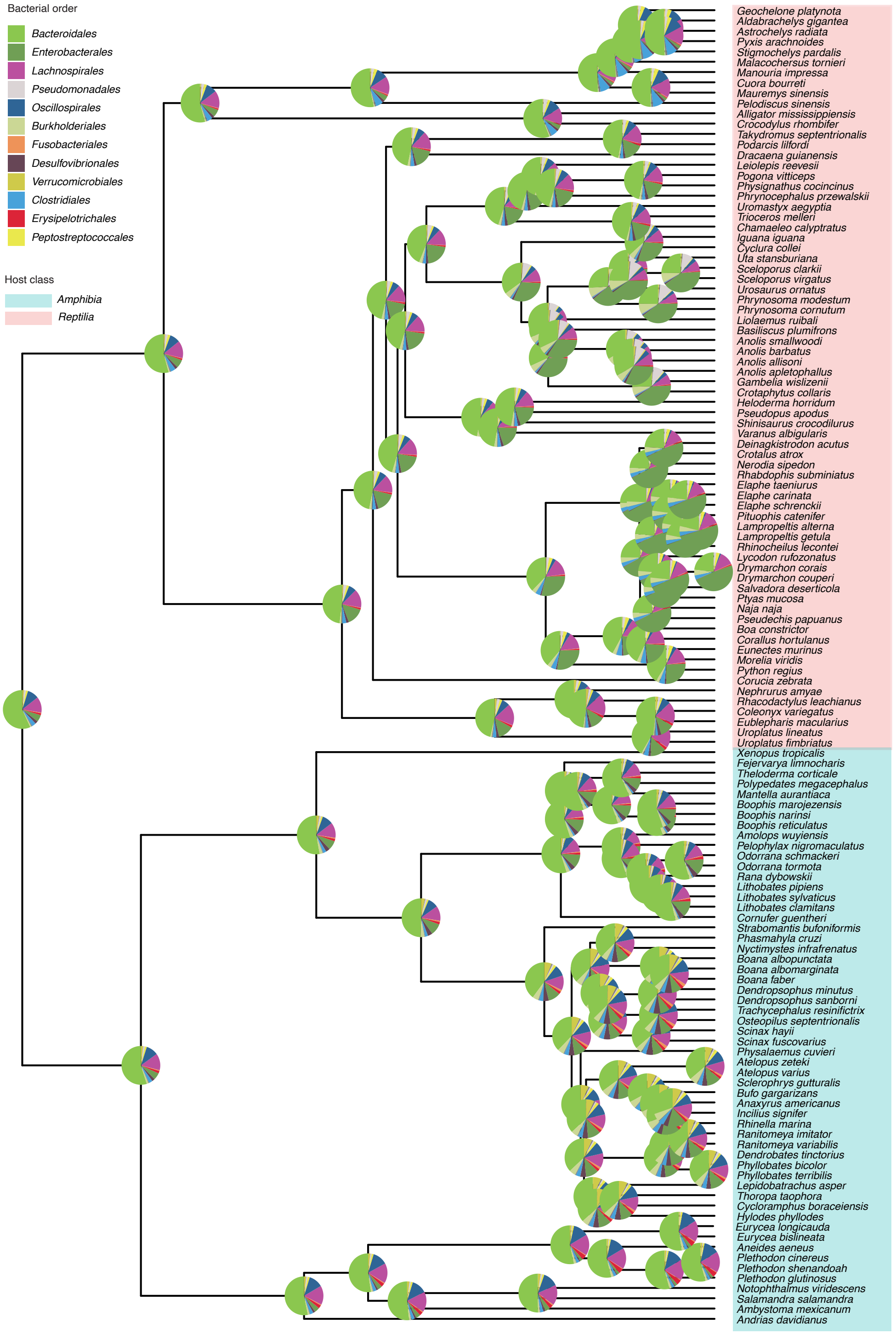

Supplementary Fig. S27 | Ancestral reconstruction of gut microbiota with phylogenetic tree of the host species and associated relative abundances of the 14 most abundant bacterial orders. The pie charts at the root and nodes of the tree show the estimated ancestral microbiota compositions (the mean of the posterior distribution at the root, and the generalized least squares estimates at the other internal nodes).

Supplementary Table S1 | The list of accession number list for the sequencing data in this study and the corresponding metadata.

Supplementary Table S2 | The amount of detectable microbial taxa at different taxonomic levels across host body sites.

	Phylum	Family	Genus
Skin	83	1,105	3,236
Oral	43	498	1,192
Stomach	40	341	751
Gut	76	900	2,490
Cloaca	58	683	1,798
Feces	46	460	1,250
Egg	57	497	950
Tail	21	182	354
Others	35	301	634

Annotation of taxa was refined to the subtaxonomic level at each classification rank according to Greengenes2 [6]. The category "Others" includes esophagus, oviduct, pelvis, lumina, and mucosa.

Supplementary Table S3 | PERMANOVA analysis testing the effects of body site, host species and their interaction on the Jaccard dissimilarity of microbial community at genus level.

	SumOfSqs	R2	F	*Pr*(>F)
BodySite	349.01	0.07	111.48	<0.01
Species	1344.98	0.28	15.39	<0.01
BodySite:Species	183.93	0.04	8.92	<0.01
Residual	2940.26	0.61		
Total	4818.19	1.00		

Supplementary Table S4 | PERMANOVA analysis testing the effects of body site, host species and their interaction on the Bray–Curtis dissimilarity of microbial community at genus level.

	SumOfSqs	R2	F	*Pr*(>F)
BodySite	358.99	0.07	106.78	<0.01
Species	1469.34	0.28	15.66	<0.01
BodySite:Species	213.62	0.04	9.65	<0.01
Residual	3157.39	0.61		
Total	5199.34	1.00		

Supplementary Table S5 | PERMANOVA analysis testing the effects of body site, host species and their interaction on the Jaccard dissimilarity of microbial community at ASV level.

	SumOfSqs	R2	F	*Pr*(>F)
BodySite	60.28	0.02	15.37	<0.01
Species	549.91	0.20	4.90	<0.01
BodySite:Species	48.43	0.02	3.01	<0.01
Residual	2027.06	0.75		
Total	2685.69	1.00		

Supplementary Table S6 | PERMANOVA analysis testing the effects of body site, host species and their interaction on the Bray–Curtis dissimilarity of microbial community at ASV level.

	SumOfSqs	R2	F	*Pr*(>F)
BodySite	81.52	0.03	23.84	<0.01
Species	760.37	0.28	7.77	<0.01
BodySite:Species	62.40	0.02	4.45	<0.01
Residual	1767.92	0.66		
Total	2672.21	1.00		

Supplementary Table S7 | PERMANOVA analysis testing the effects of body site, host species and their interaction on the unweighted UniFrac dissimilarity of microbial community at ASV level

	SumOfSqs	R2	F	*Pr*(>F)
BodySite	158.23	0.08	66.22	<0.01
Species	507.13	0.26	7.42	<0.01
BodySite:Species	43.58	0.02	4.45	<0.01
Residual	1235.35	0.64		
Total	1944.30	1.00		

Supplementary Table S8 | PERMANOVA analysis testing the effects of body site, host species and their interaction on the weighted UniFrac dissimilarity of microbial community at ASV level.

	SumOfSqs	R2	F	*Pr*(>F)
BodySite	597.28	0.29	401.76	<0.01
Species	641.33	0.31	15.08	<0.01
BodySite:Species	82.43	0.04	13.52	<0.01
Residual	768.61	0.37		
Total	2089.66	1.00		

Supplementary Table S9 | PERMANOVA analysis testing the effects of body site, host species and their interaction on the Bray–Curtis dissimilarity of microbial potential function

	SumOfSqs	R2	F	*Pr*(>F)
BodySite	38.81	0.20	195.13	<0.01
Species	51.74	0.26	9.09	<0.01
BodySite:Species	4.76	0.02	5.84	<0.01
Residual	102.84	0.52		
Total	198.15	1.00		

Supplementary Table S10 | PERMANOVA results for the effects of host species on the microbial potential functions across different body sites based on Bray–Curtis distance. The effect size has been standardized by calculating partial omega–squared.

Body site	F	parOmegaSq	*Pr*(>F)
Skin	10.26	0.32	<0.01
Oral	7.74	0.32	<0.01
Stomach	2.83	0.19	<0.01
Gut	4.83	0.24	<0.01
Cloaca	23.04	0.45	<0.01
Feces	11.41	0.42	<0.01

Supplementary Table S11 | Correlations between host phylogenetic distance and Jaccard distance of the microbial communities at genus level using Mantel test.

Body site	Pearson		Spearman	
	r	*P*	r	*P*
Skin	0.09	0.02	0.06	0.08
Oral	0.72	0.05	0.53	0.04
Stomach	0.85	<0.01	0.70	<0.01
Gut	0.15	<0.01	0.11	<0.01
Cloaca	0.41	<0.01	0.11	0.22
Feces	0.39	<0.01	0.27	0.01

Supplementary Table S12 | Correlations between host phylogenetic distance and microbial community Bray–Curtis distance of the microbial communities at genus level using Mantel test.

Body site	Pearson		Spearman	
	r	*P*	r	*P*
Skin	0.22	<0.01	0.17	<0.01
Oral	–0.19	0.74	–0.19	0.73
Stomach	0.81	0.01	0.53	0.04
Gut	0.18	<0.01	0.17	<0.01
Cloaca	0.59	<0.01	0.50	<0.01
Feces	0.35	<0.01	0.29	0.01

Supplementary Table S13 | Correlations between host phylogenetic distance and microbial community Jaccard distance of the microbial communities at ASV level using Mantel test.

Body site	Pearson		Spearman	
	r	*P*	r	*P*
Skin	0.43	<0.01	0.58	<0.01
Oral	0.76	0.25	0.74	0.25
Stomach	0.88	<0.01	0.98	<0.01
Gut	0.35	<0.01	0.31	<0.01
Cloaca	0.68	<0.01	0.72	<0.01
Feces	0.46	<0.01	0.38	<0.01

Supplementary Table S14 | Correlations between host phylogenetic distance and microbial community Bray–Curtis distance of the microbial communities at ASV level using Mantel test.

Body site	Pearson		Spearman	
	r	*P*	r	*P*
Skin	0.37	0.01	0.57	<0.01
Oral	–0.45	0.92	–0.28	0.75
Stomach	0.84	<0.01	0.97	<0.01
Gut	0.29	<0.01	0.26	<0.01
Cloaca	0.57	<0.01	0.74	<0.01
Feces	0.49	<0.01	0.45	<0.01

Supplementary Table S15 | Correlations between host phylogenetic distance and microbial community unweighted UniFrac distance of the microbial communities at ASV level using Mantel test.

Body site	Pearson		Spearman	
	r	*P*	r	*P*
Skin	0.14	<0.01	0.12	<0.01
Oral	0.60	0.25	0.49	0.25
Stomach	0.74	<0.01	0.75	<0.01
Gut	0.19	<0.01	0.15	<0.01
Cloaca	0.66	<0.01	0.49	<0.01
Feces	0.46	<0.01	0.35	<0.01

Supplementary Table S16 | Correlations between host phylogenetic distance and microbial community weighted UniFrac distance of the microbial communities at ASV level using Mantel test.

Body site	Pearson		Spearman	
	r	*P*	r	*P*
Skin	0.19	<0.01	0.19	<0.01
Oral	–0.41	0.92	–0.68	0.92
Stomach	0.48	0.05	0.41	0.10
Gut	0.09	<0.01	0.08	<0.01
Cloaca	0.54	<0.01	0.68	<0.01
Feces	0.05	0.38	0.13	0.15

Supplementary Table S17 | Results of testing for the congruency of topologies between host phylogeny and microbial dendrogram based on Jaccard distance at microbial genus level using Robonson–Foulds (RF) and Matching Cluster (MC) metrics.

Body site	Robinson–Foulds		Matching Cluster	
	nRF	*P*	nMC	*P*
Skin	0.96	<0.01	0.79	<0.01
Oral	0.75	0.27	0.38	0.08
Stomach	0.88	0.24	0.42	0.04
Gut	0.95	<0.01	0.83	0.77
Cloaca	0.80	<0.01	0.52	0.07
Feces	0.92	<0.01	0.82	0.97

Nomalized Robinson–Foulds (nRF) and normalized Matching Cluster (nMC) metrics were determined following the methods by Brooks et al. [18] . Nomalized metrics (nRF and nMC) scale from 0.0 (complete congruence) to 1.0 (complete incongruence).

Supplementary Table S18 | Results of testing for the congruency of topologies between host phylogeny and microbial dendrogram based on Bray–Curtis distance at microbial genus level using Robonson–Foulds (RF) and Matching Cluster (MC) metrics.

Body site	Robinson–Foulds		Matching Cluster	
	nRF	*P*	nMC	*P*
Skin	0.96	<0.01	0.84	0.02
Oral	1.00	1.00	0.69	0.79
Stomach	0.88	0.23	0.42	0.02
Gut	0.98	<0.01	0.80	0.03
Cloaca	0.73	<0.01	0.38	<0.01
Feces	0.92	<0.01	0.71	0.24

Nomalized Robinson–Foulds (nRF) and normalized Matching Cluster (nMC) metrics were determined following the methods by Brooks et al. [18]. Nomalized metrics

(nRF and nMC) scale from 0.0 (complete congruence) to 1.0 (complete incongruence).

Supplementary Table S19 | Results of testing for the congruency of topologies between host phylogeny and microbial dendrogram based on Jaccard distance at microbial ASV level using Robonson–Foulds (RF) and Matching Cluster (MC) metrics.

Body site	Robinson–Foulds		Matching Cluster	
	nRF	*P*	nMC	*P*
Skin	0.97	<0.01	0.77	<0.01
Oral	0.50	0.33	0.33	0.16
Stomach	0.40	0.01	0.15	<0.01
Gut	0.94	<0.01	0.64	<0.01
Cloaca	0.58	<0.01	0.32	<0.01
Feces	0.78	<0.01	0.58	0.04

Nomalized Robinson–Foulds (nRF) and normalized Matching Cluster (nMC) metrics were determined following the methods by Brooks et al. [18]. Nomalized metrics (nRF and nMC) scale from 0.0 (complete congruence) to 1.0 (complete incongruence).

Supplementary Table S20 | Results of testing for the congruency of topologies between host phylogeny and microbial dendrogram based on Bray–Curtis distance at microbial ASV level using Robonson–Foulds (RF) and Matching Cluster (MC) metrics.

Body site	Robinson–Foulds		Matching Cluster	
	nRF	*P*	nMC	*P*
Skin	0.96	<0.01	0.81	<0.01
Oral	1.00	1.00	0.50	0.66
Stomach	0.40	0.01	0.20	<0.01
Gut	0.94	<0.01	0.81	0.01
Cloaca	0.67	<0.01	0.51	0.14
Feces	0.83	<0.01	0.58	0.04

Nomalized Robinson–Foulds (nRF) and normalized Matching Cluster (nMC) metrics were determined following the methods by Brooks et al. [18]. Nomalized metrics (nRF and nMC) scale from 0.0 (complete congruence) to 1.0 (complete incongruence).

Supplementary Table S21 | Results of testing for the congruency of topologies between host phylogeny and microbial dendrogram based on unweighted UniFrac distance at microbial ASV level using Robonson–Foulds (RF) and Matching Cluster (MC) metrics.

Body site	Robinson–Foulds		Matching Cluster	
	nRF	*P*	nMC	*P*
Skin	0.98	0.02	0.86	0.06
Oral	0.50	0.33	0.33	0.16
Stomach	0.60	0.06	0.45	0.1
Gut	0.96	<0.01	0.73	<0.01
Cloaca	0.67	<0.01	0.40	<0.01
Feces	0.83	<0.01	0.49	<0.01

Nomalized Robinson–Foulds (nRF) and normalized Matching Cluster (nMC) metrics were determined following the methods by Brooks et al. [18]. Nomalized metrics (nRF and nMC) scale from 0.0 (complete congruence) to 1.0 (complete incongruence).

Supplementary Table S22 | Results of testing for the congruency of topologies between host phylogeny and microbial dendrogram based on weighted UniFrac distance at microbial ASV level using Robonson–Foulds (RF) and Matching Cluster (MC) metrics.

Body site	Robinson–Foulds		Matching Cluster	
	nRF	*P*	nMC	*P*
Skin	0.95	<0.01	0.79	<0.01
Oral	1.00	1.00	1.00	1.00
Stomach	0.80	0.24	0.45	0.09
Gut	0.97	<0.01	1.00	1.00
Cloaca	0.50	<0.01	0.29	<0.01
Feces	0.86	<0.01	0.63	0.80

Nomalized Robinson–Foulds (nRF) and normalized Matching Cluster (nMC) metrics were determined following the methods by Brooks et al. [18]. Nomalized metrics (nRF and nMC) scale from 0.0 (complete congruence) to 1.0 (complete incongruence).

Supplementary Table S23 | Correlations between amphibian phylogeny and fecal microbial communities at ASV level using Mantel test.

Beta diversity indices	Pearson		Spearman	
	r	*P*	r	*P*
Jaccard	0.59	0.01	0.19	0.08
Bray–Curtis	0.56	0.03	0.13	0.12
Unweighted UniFrac	0.78	<0.01	0.72	<0.01
Weighted UniFrac	0.67	0.01	0.57	0.02

Supplementary Table S24 | Correlations between amphibian phylogeny and gut microbial communities at ASV level using Mantel test.

Beta diversity indices	Pearson		Spearman	
	r	*P*	r	*P*
Jaccard	0.24	<0.01	0.29	<0.01
Bray–Curtis	0.24	<0.01	0.29	<0.01
Unweighted UniFrac	0.06	0.12	0.03	0.30
Weighted UniFrac	0.02	0.40	<0.01	0.47

Supplementary Table S25 | Correlations between amphibian phylogeny and skin microbial communities at ASV level using Mantel test.

Beta diversity indices	Pearson		Spearman	
	r	*P*	r	*P*
Jaccard	0.19	<0.01	0.28	<0.01
Bray–Curtis	0.18	<0.01	0.24	<0.01
Unweighted UniFrac	0.16	<0.01	0.16	<0.01
Weighted UniFrac	0.13	0.02	0.16	<0.01

Supplementary Table S26 | Correlations between reptile phylogeny and fecal microbial communities at ASV level using Mantel test.

Beta diversity indices	Pearson		Spearman	
	r	*P*	r	*P*
Jaccard	0.43	<0.01	0.40	<0.01
Bray–Curtis	0.46	<0.01	0.48	<0.01
Unweighted UniFrac	0.49	<0.01	0.40	<0.01
Weighted UniFrac	0.24	0.12	0.32	0.02

Supplementary Table S27 | Correlations between reptile phylogeny and gut microbial communities at ASV level using Mantel test.

Beta diversity indices	Pearson		Spearman	
	r	*P*	r	*P*
Jaccard	0.21	<0.01	0.11	0.03
Bray–Curtis	0.19	<0.01	0.11	0.04
Unweighted UniFrac	0.11	0.04	0.08	0.10
Weighted UniFrac	0.08	0.08	0.08	0.10

Supplementary Table S28 | Correlations between reptile phylogeny and skin microbial communities at ASV level using Mantel test.

Beta diversity indices	Pearson		Spearman	
	r	*P*	r	*P*
Jaccard	0.41	<0.01	0.39	<0.01
Bray–Curtis	0.33	<0.01	0.42	<0.01
Unweighted UniFrac	0.33	<0.01	0.24	0.02
Weighted UniFrac	-0.07	0.70	-0.08	0.75

Supplementary Table S29 | Results of testing topological congruence between amphibian phylogeny and fecal microbial dendrograms at the ASV level using Robinson–Foulds (RF) and Matching Cluster (MC) metrics.

Beta diversity indices	Robinson–Foulds		Matching Cluster	
	nRF	*P*	nMC	*P*
Jaccard	0.50	0.08	0.54	0.30
Bray–Curtis	0.75	0.28	0.62	0.48
Unweighted UniFrac	0.0	<0.01	0.0	<0.01
Weighted UniFrac	0.75	0.28	0.38	0.08

Nomalized Robinson–Foulds (nRF) and normalized Matching Cluster (nMC) metrics were determined following the methods by Brooks et al. [18]. Nomalized metrics (nRF and nMC) scale from 0.0 (complete congruence) to 1.0 (complete incongruence).

Supplementary Table S30 | Results of testing topological congruence between amphibian phylogeny and gut microbial dendrograms at the ASV level using Robinson–Foulds (RF) and Matching Cluster (MC) metrics.

Beta diversity indices	Robinson–Foulds		Matching Cluster	
	nRF	*P*	nMC	*P*
Jaccard	0.89	<0.01	0.65	<0.01
Bray–Curtis	0.89	<0.01	0.64	<0.01
Unweighted UniFrac	0.91	<0.01	0.75	0.14
Weighted UniFrac	0.96	0.02	0.78	0.24

Nomalized Robinson–Foulds (nRF) and normalized Matching Cluster (nMC) metrics were determined following the methods by Brooks et al. [18]. Nomalized metrics (nRF and nMC) scale from 0.0 (complete congruence) to 1.0 (complete incongruence).

Supplementary Table S31 | Results of testing topological congruence between amphibian phylogeny and skin microbial dendrograms at the ASV level using Robinson–Foulds (RF) and Matching Cluster (MC) metrics.

Beta diversity indices	Robinson–Foulds		Matching Cluster	
	nRF	*P*	nMC	*P*
Jaccard	0.98	0.02	0.79	<0.01

Bray–Curtis	0.96	<0.01	0.88	0.43
Unweighted UniFrac	0.98	0.02	0.78	<0.01
Weighted UniFrac	0.95	<0.01	0.70	<0.01

Nomalized Robinson–Foulds (nRF) and normalized Matching Cluster (nMC) metrics were determined following the methods by Brooks et al. [18]. Nomalized metrics (nRF and nMC) scale from 0.0 (complete congruence) to 1.0 (complete incongruence).

Supplementary Table S32 | Results of testing topological congruence between reptile phylogeny and fecal microbial dendrograms at the ASV level using Robinson–Foulds (RF) and Matching Cluster (MC) metrics.

Beta diversity indices	Robinson–Foulds		Matching Cluster	
	nRF	*P*	nMC	*P*
Jaccard	0.82	<0.01	0.56	0.02
Bray–Curtis	0.82	<0.01	0.49	0.02
Unweighted UniFrac	0.82	<0.01	0.45	<0.01
Weighted UniFrac	0.88	0.02	0.55	0.05

Nomalized Robinson–Foulds (nRF) and normalized Matching Cluster (nMC) metrics were determined following the methods by Brooks et al. [18]. Nomalized metrics (nRF and nMC) scale from 0.0 (complete congruence) to 1.0 (complete incongruence).

Supplementary Table S33 | Results of testing topological congruence between reptile phylogeny and gut microbial dendrograms at the ASV level using Robinson–Foulds (RF) and Matching Cluster (MC) metrics.

Beta diversity indices	Robinson–Foulds		Matching Cluster	
	nRF	*P*	nMC	*P*
Jaccard	1.00	1.00	0.67	<0.01
Bray–Curtis	0.98	0.18	0.75	<0.01
Unweighted UniFrac	1.00	1.00	0.77	0.02
Weighted UniFrac	1.00	1.00	0.83	0.14

Nomalized Robinson–Foulds (nRF) and normalized Matching Cluster (nMC) metrics were determined following the methods by Brooks et al. [18]. Nomalized metrics (nRF and nMC) scale from 0.0 (complete congruence) to 1.0 (complete incongruence).

Supplementary Table S34 | Results of testing topological congruence between reptile phylogeny and skin microbial dendrograms at the ASV level using Robinson–Foulds (RF) and Matching Cluster (MC) metrics.

Beta diversity indices	Robinson–Foulds		Matching Cluster	
	nRF	*P*	nMC	*P*
Jaccard	0.90	0.04	0.52	0.60
Bray–Curtis	0.95	0.26	0.86	1.0
Unweighted UniFrac	1.0	1.0	0.75	0.98
Weighted UniFrac	0.95	0.27	0.64	0.90

Nomalized Robinson–Foulds (nRF) and normalized Matching Cluster (nMC) metrics were determined following the methods by Brooks et al. [18]. Nomalized metrics (nRF and nMC) scale from 0.0 (complete congruence) to 1.0 (complete incongruence).

Supplementary Table S35 | Summary of Pagel's lambda using ABDOMEN (A Brownian moDel Of Microbiota EvolutioN).

Host group	Body site	Pagel's lambda	*P* value	Reference
Mammals	Feces	0.65	<0.01	[13]
	Feces	0.41	0.01	[23]
Herpetofauna	Skin	0.13	<0.01	This study
	Gut	0.18	0.01	This study
	Feces	0.21	0.01	This study

Pagel's lambda estimates close to 1 indicate that an untransformed tree explains the data well, reflecting strong phylosymbiosis, whereas values near 0 suggest weak or no explanatory power of the tree and thus minimal or absent phylosymbiosis.

Supplementary Table S36 | Summary of the correlation between host phylogeny and gut microbiota at ASV level in fish and mammals using Mantel test.

Host group	Result	Reference
Fish	$r = 0.1265$, $P > 0.05$	[24]
	$r = 0.18$, $P < 0.05$	[25]
	$r = 0.26$, $P = 0.009$	[26]
	$r = 0.306$, $P = 0.008$	[27]
	$r = 0.4231$, $P = 0.003$	[28]
Mammal	$r = 0.459$, $P = 0.009$	[29]
	$r = 0.77$, $P < 0.0001$	[30]
	$r = 0.8025$, $P < 0.05$	[31]
	$r = 0.466–0.746$, $P \leq 0.05$	[15]
	$r = 0.68$, $P = 0.001$	[32]
	$r = 0.56$, $P \leq 0.001$	[33]
	$r = 0.83$, $P < 0.001$	[34]

Supplementary Table S37 | Summary of the congruency of topologies between host phylogeny and microbial dendrogram in mammals using Robinson–Foulds (nRF) or Matching Cluster (nMC) metrics in previous studies.

Body site	Host order	Robinson–Foulds		Matching Cluster		Reference
		nRF	*P*	nMC	*P*	
Skin	Mutiple orders	0.97	>0.05	0.98	>0.05	[35]
	Perissodactyla	0.33	0.009	0.33	0.002	[35]
	Artiodactyla	0.71	0.03	0.38	0.001	[35]
	Carnivora	0.83	0.3	0.79	0.27	[35]
Stomach	Rodentia	0.58	0.005	0.32	<0.001	[36]
Gut	Rodentia	0.52–0.60	<0.04	0.28–0.33	<0.005	[36]
Feces	Mutiple orders	0.33	<0.001	NA	NA	[34]
	Mutiple orders	0.333	0.0003	NA	NA	[37]
	Rodentia	0.5	0.056	0.25	0.006	[18]
	Primates	0.33	0.003	0.23	0.001	[18]
	Artiodactyla	0.5	0.0535	0.4	0.0461	[31]
	Rodentia	0.44	0.005	0.33	<0.001	[36]

Nomalized Robinson–Foulds (nRF) and normalized Matching Cluster (nMC) metrics were determined following the methods by Brooks et al. [18]. Nomalized metrics (nRF and nMC) scale from 0.0 (complete congruence) to 1.0 (complete incongruence).

References

1. Li J, Gao Y, Shu G et al. Hmicrodb: A comprehensive database of herpetofaunal microbiota with a focus on host phylogeny, physiological traits, and environment factors. Molecular Ecology Resources.25:e14046 https://doi.org/https://doi.org/10.1111/1755-0998.14046

2. Meyer M, Kircher M. Illumina sequencing library preparation for highly multiplexed target capture and sequencing. Cold Spring Harbor protocols. 2010;2010:pdb.prot5448 https://doi.org/10.1101/pdb.prot5448

3. Margulies M, Egholm M, Altman W et al. Genome sequencing in microfabricated high-density picolitre reactors. Nature. 2005;437:376-80 https://doi.org/10.1038/nature03959

4. Chen S, Zhou Y, Chen Y et al. Fastp: An ultra-fast all-in-one fastq preprocessor. Bioinformatics. 2018;34:i884-i90 https://doi.org/10.1093/bioinformatics/bty560

5. Bolyen E, Rideout JR, Dillon MR et al. Reproducible, interactive, scalable and extensible microbiome data science using qiime 2. Nature Biotechnology. 2019;37:852-57 https://doi.org/10.1038/s41587-019-0209-9

6. McDonald D, Jiang Y, Balaban M et al. Greengenes2 unifies microbial data in a single reference tree. Nature Biotechnology. 2024;42:813 https://doi.org/10.1038/s41587-023-01845-1

7. Kurilshikov A, Medina-Gomez C, Bacigalupe R et al. Large-scale association analyses identify host factors influencing human gut microbiome composition. Nat Genet. 2021;53:156-65 https://doi.org/10.1038/s41588-020-00763-1

8. Lin Q, Dorsett Y, Mirza A et al. Meta-analysis identifies common gut microbiota associated with multiple sclerosis. Genome Med. 2024;16:94 https://doi.org/10.1186/s13073-024-01364-x

9. Douglas GM, Maffei VJ, Zaneveld JR et al. Picrust2 for prediction of metagenome functions. Nature Biotechnology. 2020;38:685-88 https://doi.org/10.1038/s41587-020-0548-6

10. Null RCTR, Team R, Null RCT et al. R: A language and environment for statistical computing. Computing. 2011;1:12-21

11. Dixon P. Vegan, a package of r functions for community ecology. Journal of Vegetation Science. 2003;14:927-30 https://doi.org/10.1111/j.1654-1103.2003.tb02228.x

12. Lim SJ, Bordenstein SR. An introduction to phylosymbiosis. Proc Biol Sci. 2020;287:20192900 https://doi.org/10.1098/rspb.2019.2900

13. Perez-Lamarque B, Sommeria-Klein G, Duret L et al. Phylogenetic comparative approach reveals evolutionary conservatism, ancestral composition, and integration of vertebrate gut microbiota. Molecular Biology and Evolution. 2023;40:msad144 https://doi.org/10.1093/molbev/msad144

14. Kumar S, Suleski M, Craig JM et al. Timetree 5: An expanded resource for species divergence times. Molecular Biology and Evolution. 2022;39:msac174 https://doi.org/10.1093/molbev/msac174

15. Zhang XY, Khakisahneh S, Liu W et al. Phylogenetic signal in gut microbial community rather than in rodent metabolic traits. Natl Sci Rev. 2023;10:nwad209 https://doi.org/10.1093/nsr/nwad209

16. Doane M, Morris M, Papudeshi B et al. The skin microbiome of elasmobranchs follows phylosymbiosis, but in teleost fishes, the microbiomes converge. Microbiome. 2020;8:93 https://doi.org/10.1186/s40168-020-00840-x

17. Derek HO, Jason CD, Powell W et al. Fsa: Simple fisheries stock assessment methods. 2025 https://doi.org/10.32614/CRAN.package.FSA

18. Brooks AW, Kohl KD, Brucker RM et al. Phylosymbiosis: Relationships and functional effects of microbial communities across host evolutionary history. PLoS Biol. 2016;14:e2000225 https://doi.org/10.1371/journal.pbio.2000225

19. Bogdanowicz D, Giaro K. On a matching distance between rooted phylogenetic trees. International Journal of Applied Mathematics and Computer Science. 2013;23:669 - 84 https://doi.org/10.2478/amcs-2013-0050

20. Robinson DF, Foulds LR. Comparison of phylogenetic trees. Mathematical Biosciences. 1981;53:131-47 https://doi.org/10.1016/0025-5564(81)90043-2

21. Team SD. Rstan: The r interface to stan. 2025

22. Pagel M. Inferring the historical patterns of biological evolution. Nature. 1999;401:877-84 https://doi.org/10.1038/44766

23. Kujawska M, Seki D, Chalklen L et al. Host-specific microbiome and genomic signatures in bifidobacterium reveal co-evolutionary and functional adaptations across diverse animal hosts. Cell Host Microbe. 2025;33:1502-17 e13 https://doi.org/10.1016/j.chom.2025.08.008

24. Soh M, Tay YC, Lee CS et al. The intestinal digesta microbiota of tropical marine fish is largely uncultured and distinct from surrounding water microbiota. npj Biofilms and Microbiomes. 2024;10:11 https://doi.org/10.1038/s41522-024-00484-x

25. Sadeghi J, Chaganti SR, Johnson TB et al. Host species and habitat shape fish-associated bacterial communities: Phylosymbiosis between fish and their microbiome. Microbiome. 2023;11:258 https://doi.org/10.1186/s40168-023-01697-6

26. Lilli G, Sirot C, Campbell H et al. Geographic origin and host's phylogeny are predictors of the gut mucosal microbiota diversity and composition in mediterranean scorpionfishes (*scorpaena* spp.). Frontiers in Marine Science. 2023;10:1286706 https://doi.org/10.3389/fmars.2023.1286706

27. Minich JJ, Härer A, Vechinski J et al. Host biology, ecology and the environment influence microbial biomass and diversity in 101 marine fish species. Nature Communications. 2022;13:6978 https://doi.org/10.1038/s41467-022-34557-2

28. Han GH, Yu J, Kang MJ et al. Phylosymbiosis in seven wild fish species collected off the southern coast of korea: Skin microbiome most strongly reflects evolutionary pressures. Microbial Ecology. 2024;87:153 https://doi.org/10.1007/s00248-024-02467-z

29. Gregor R, Probst M, Eyal S et al. Mammalian gut metabolomes mirror microbiome composition and host phylogeny. The ISME Journal. 2022;16:1262-74

https://doi.org/10.1038/s41396-021-01152-0

30. Rojas CA, Ramírez-Barahona S, Holekamp KE et al. Host phylogeny and host ecology structure the mammalian gut microbiota at different taxonomic scales. Animal Microbiome. 2021;3:33 https://doi.org/10.1186/s42523-021-00094-4

31. Li J, Zhan S, Liu X et al. Divergence of fecal microbiota and their associations with host phylogeny in *Cervinae*. Front Microbiol. 2018;9:1823 https://doi.org/10.3389/fmicb.2018.01823

32. Donohue ME, Rowe AK, Kowalewski E et al. Significant effects of host dietary guild and phylogeny in wild lemur gut microbiomes. ISME Communications. 2022;2:33 https://doi.org/10.1038/s43705-022-00115-6

33. Brown B, Goheen J, Newsome S et al. Host phylogeny and functional traits differentiate gut microbiomes in a diverse natural community of small mammals. Molecular Ecology. 2023;32:2320-34 https://doi.org/10.1111/mec.16874

34. Perofsky AC, Lewis RJ, Meyers LA. Terrestriality and bacterial transfer: A comparative study of gut microbiomes in sympatric malagasy mammals. The ISME Journal. 2019;13:50-63 https://doi.org/10.1038/s41396-018-0251-5

35. Ross AA, Muller KM, Weese JS et al. Comprehensive skin microbiome analysis reveals the uniqueness of human skin and evidence for phylosymbiosis within the class mammalia. Proc Natl Acad Sci U S A. 2018;115:E5786-E95 https://doi.org/10.1073/pnas.1801302115

36. Kohl KD, Dearing MD, Bordenstein SR. Microbial communities exhibit host species distinguishability and phylosymbiosis along the length of the gastrointestinal tract. Mol Ecol. 2018;27:1874-83 https://doi.org/10.1111/mec.14460

37. Trevelline BK, Moeller AH. Robustness of mammalian gut microbiota to humanization in captivity. Front Ecol Evol. 2022;9:785089 https://doi.org/10.3389/fevo.2021.785089
